# Supplementary figures and images for: Mutual repression between JNK/AP-1 and JAK/STAT stratifies senescent and proliferative cell behaviors during tissue regeneration
Source: PLoS Biol. 2023 May 30;21(5):e3001665. doi: 10.1371/journal.pbio.3001665 (PMC10228795; doi:10.1371/journal.pbio.3001665)

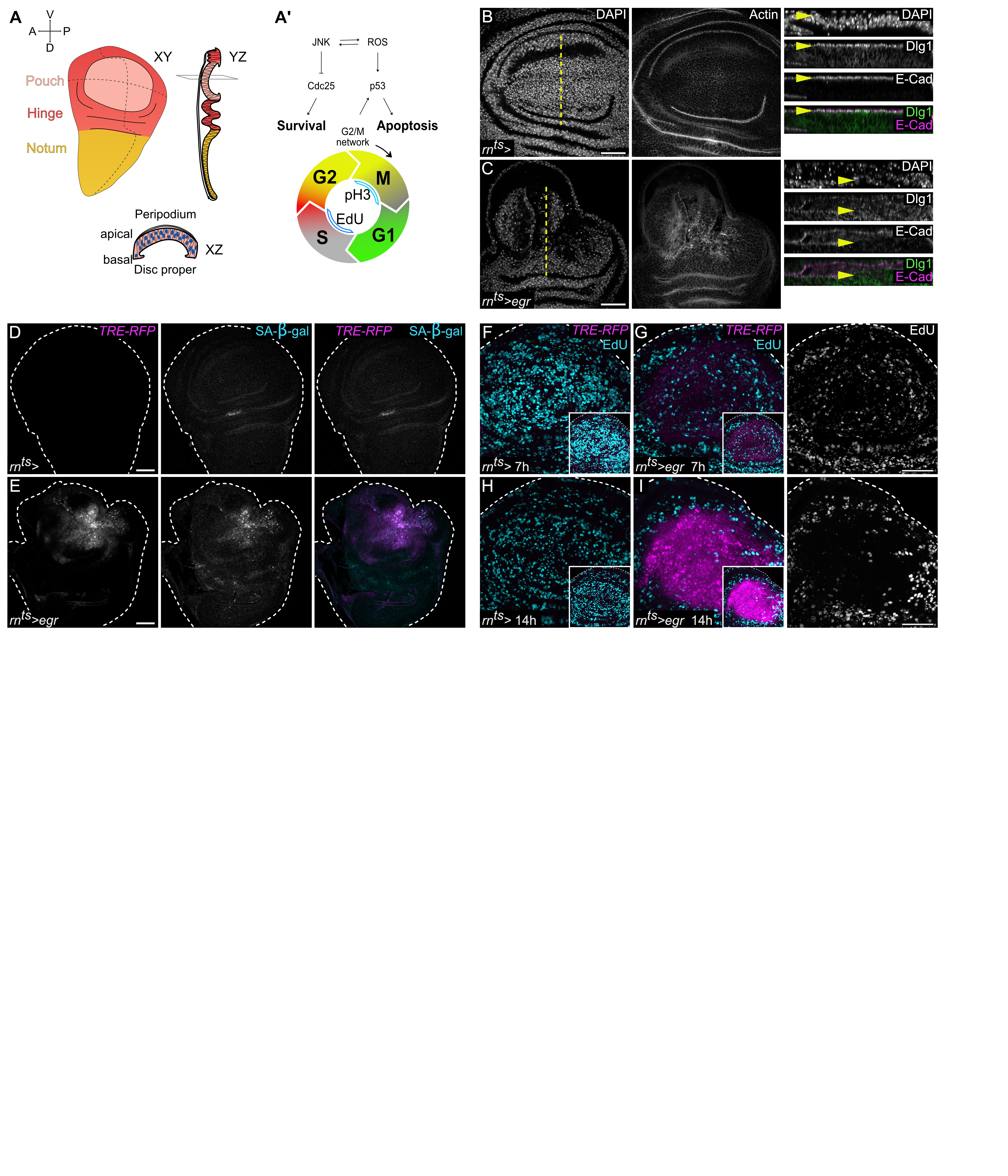

Supplement: S1 Fig — (A) Schematic of a central XY, YZ, and XZ section through a third instar wing imaginal disc epithelium. Different shades represent the pouch, hinge and notum domains. Dashed lines represent the anterior-posterior (A/P) and dorsal-ventral (D/V) compartment boundaries. The YZ section visualizes the pseudostratified monolayer organization of the disc proper, and the overlying peripodial cell layer. XZ section visualizes the apical to basal orientation of epithelial cells in the wing pouch. (A’) Damage-induced JNK/AP-1 signaling mediates survival or apoptosis through control of cell cycle progression [29,36]. JNK also controls production of ROS, which is thought to act as signaling molecule but also induces oxidative damage, which could trigger p53-dependent apoptosis. However, p53 is activated by the G2/M kinase Cdk1 and thus competent to mediate damage-induced apoptosis in G1. (B, C) XY view of control (B) and egr-expressing (C) discs after 24 h of expression stained for filamentous Actin (phalloidin). XZ sections through the tissue were visualized along dotted yellow lines. Immunostaining for E-cadherin (E-Cad, magenta, adherens junction marker) and Discs large (Dlg, green, basal polarity marker) reveals reduced cell adhesion and cell polarity, and thus barrier integrity, in an egr-expressing disc when compared to a control disc. A total of n = 3, control and n = 3, egr-expressing discs were evaluated. (D, E) A control (D) and egr-expressing (E) discs after 24 h of expression assessed for senescence-associated beta galactosidase (SA-β-gal, cyan) activity using a SA-β-gal assay. Discs also express the JNK/AP-1 activity reporter TRE-RFP (magenta). Images show TRE-RFP overlay with the same discs as shown in Fig 1. A total of n = 23, 24 h control and n = 22, 24 h egr-expressing discs were evaluated from N = 2 independent experiments. (F-I) A time-course analysis of JNK/AP-1 reporter TRE-RFP (magenta) and EdU incorporation to detect DNA replication activity (EdU, Cyan) in [file pbio.3001665.s001.tiff]

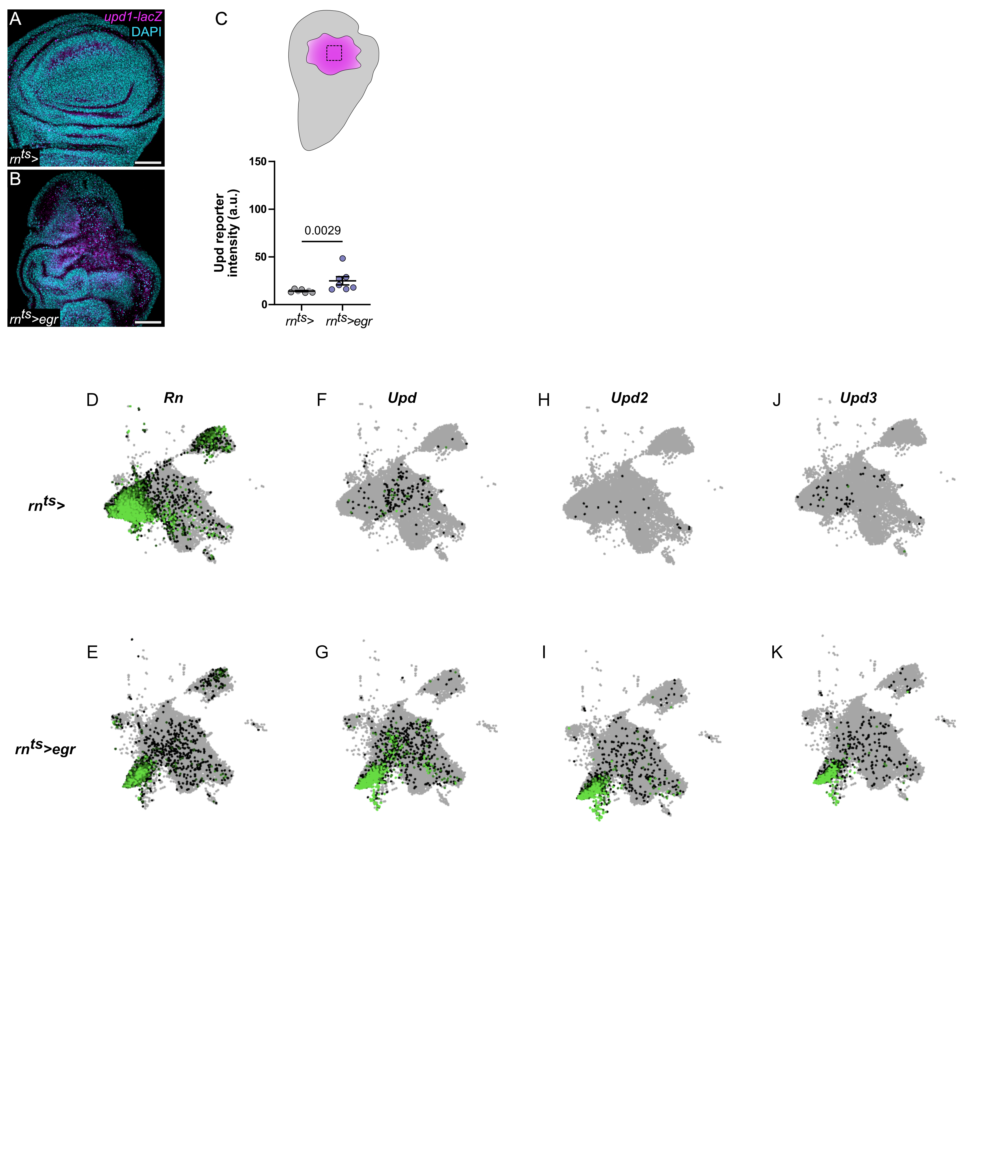

Supplement: S2 Fig — (A-C) A control (A) and egr-expressing (B) discs assessed for upd-lacZ cytokine reporter up-regulation after 24 h of expression. Graph (C) displays mean ± SEM for n = 7, control discs; n = 7, egr-expressing discs. Mann–Whitney U test was performed to test for statistical significance. (D, E) UMAP plots of control (top row) and egr-expressing (bottom row) discs analysed after 24 h of expression by single-cell RNA-Seq technology (see [72] for details). Rn-expression marks the rn-GAL4-positive cell population in control discs, and those that survive in egr-expressing discs. Induction of upd1-3 transcripts in the rn-positive cell population of egr-expressing discs (bottom row) can be clearly detected. Plots were generated using the Scope Wing Atlas [72]. Source data for quantifications provided in S1 File. Maximum projections of multiple confocal sections are shown in A and B. Discs were stained with DAPI to visualize nuclei. Scale bars: 50 μm. (TIFF) [file pbio.3001665.s002.tiff]

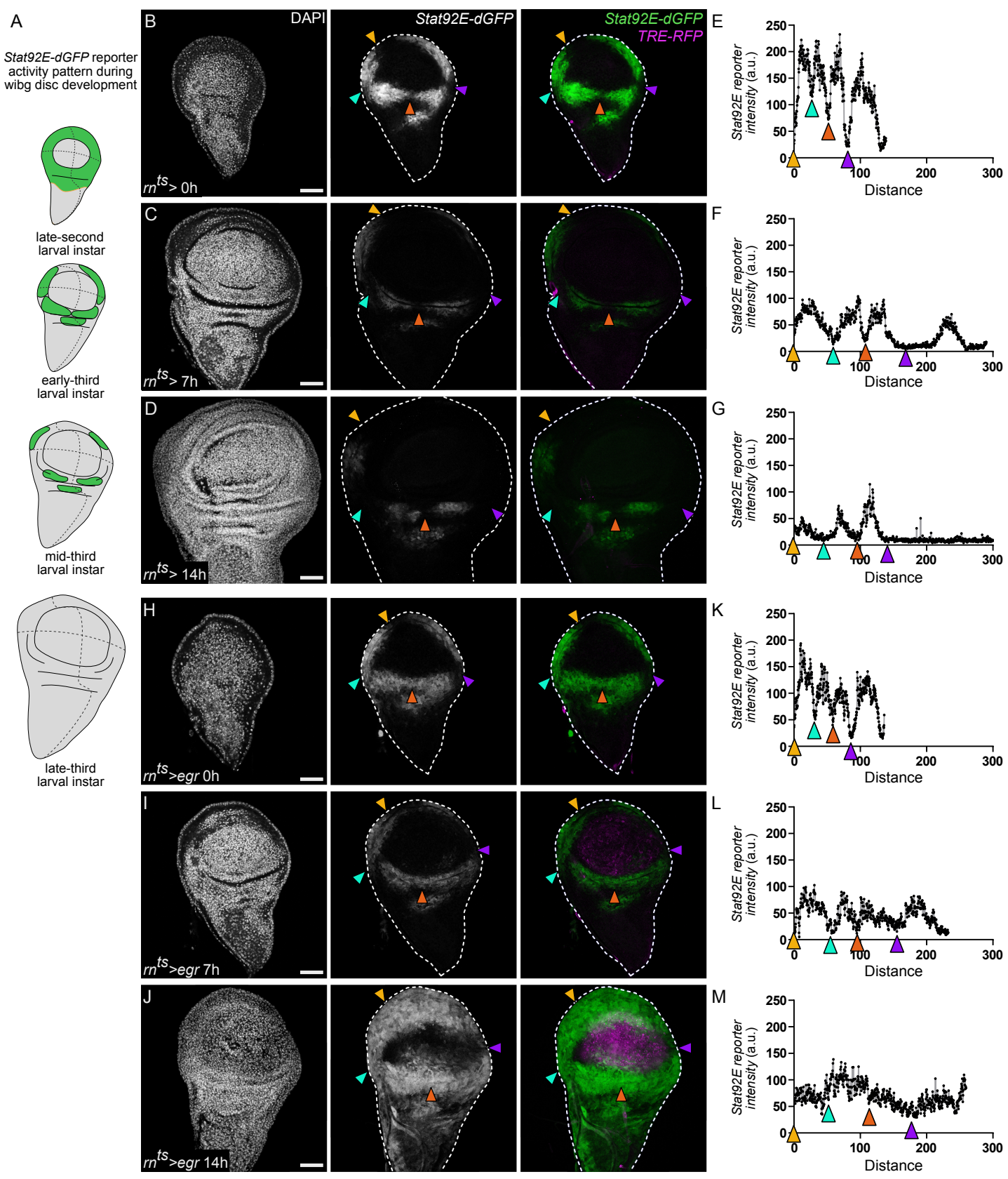

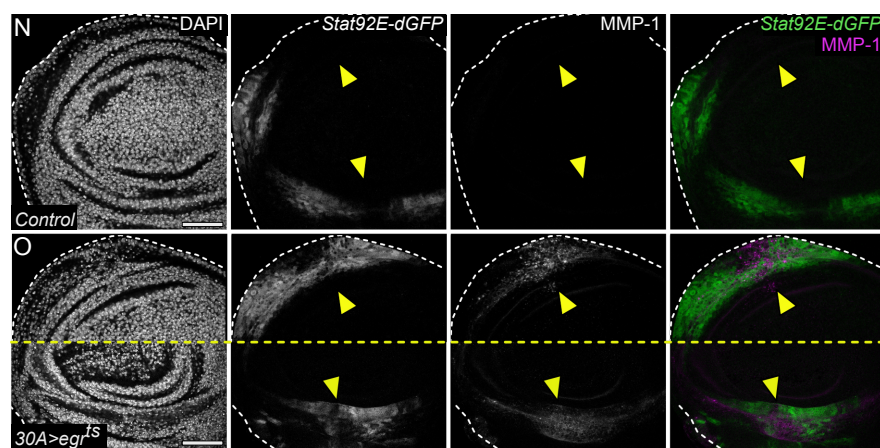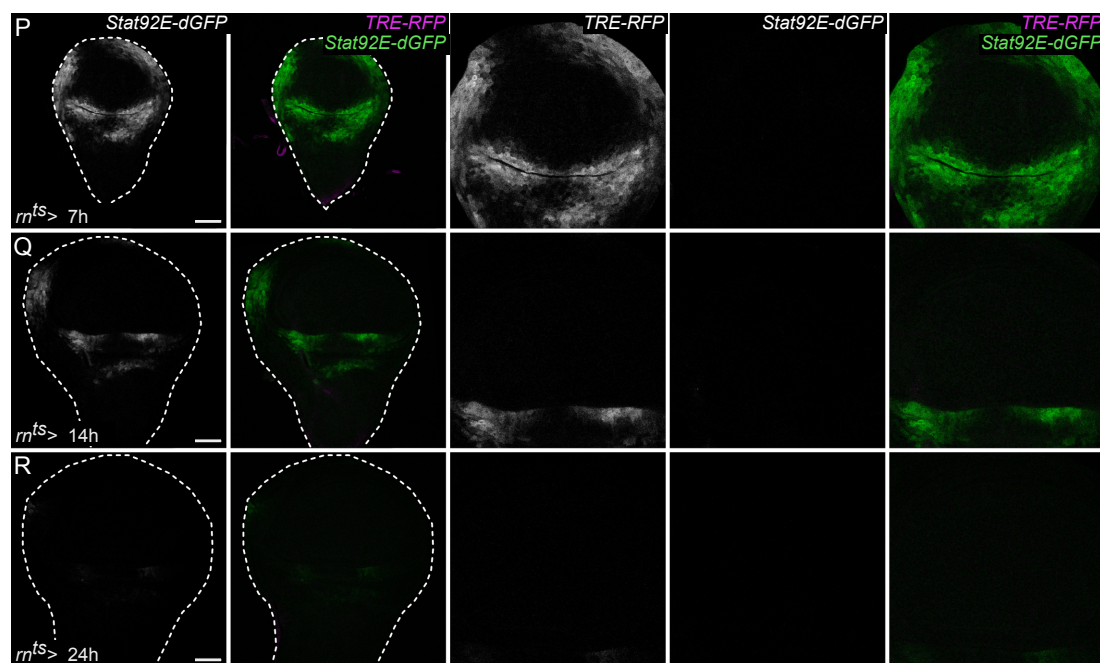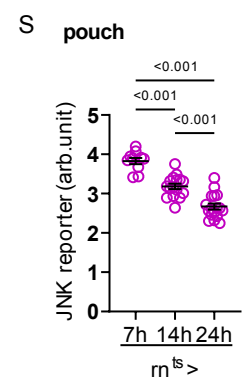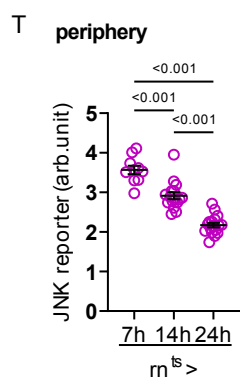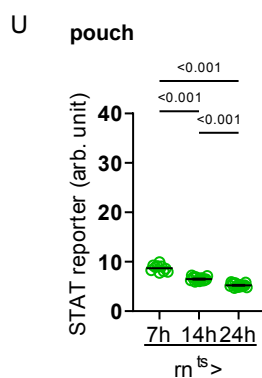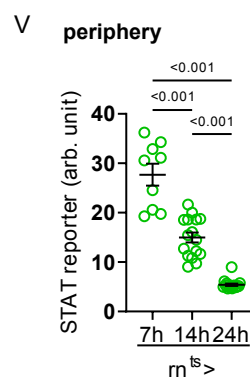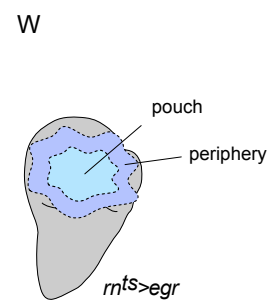

Supplement: S3 Fig — (A) Schematic showing dynamic patterning of the JAK/STAT signaling reporter (Stat92E-dGFP, green) activity in developing, undamaged wing imaginal discs. Note the gradual loss of hinge-specific patterns from mid to late third larval instar stages. (B-D) Undamaged control discs at 0 h (B), 7 h (C), and 14 h (D) of inductive temperature shift to 30°C. Disc also express the JNK/AP-1 reporter TRE-RFP (magenta) and the dynamic JAK/STAT reporter Stat92E-dGFP (green and grey). (H-J) Wing discs after 0 h (H), 7 h (I), and 14 h (J) of egr-expression in the pouch using rn-GAL4. Disc also express the JNK/AP-1 reporter TRE-RFP (magenta) and the dynamic JAK/STAT reporter Stat92E-dGFP (green and grey). Increasing JNK/AP-1 reporter activity is seen in the pouch and increasing JAK/STAT reporter is seen in the pouch and hinge from 7 h to 14 h of egr-expression. (E-G, K-M) Stat92E-dGFP reporter fluorescence intensity, traced along the hinge of control and egr-expressing discs at 0 h (E, K), 7 h (F, L), and 14 h (G, M) of egr-expression. Colored arrowheads indicate similar positions along hinge regions where JAK/STAT reporter activity is developmentally low but progressively increasing in egr-expressing discs (compare G and M). Also note that the average JAK/STAT reporter activity is higher at 14 h of egr-expression than in controls. Graphs display hinge traces from 1 representative disc. A total of n = 7, 0 h; n = 12, 7 h; and n = 5, 14 h control discs and n = 7, 0 h; n = 22, 7 h; and n = 19, 14 h egr-expressing discs were evaluated from N = 2 independent experiments. (N, O) Expression of egr using the 30A-GAL4 hinge driver. Discs were also expressing the Stat92E-dGFP (green) reporter and efficiency of JNK/AP1-activation was assessed by staining for the JNK-target MMP-1 (magenta). Please note how egr-expression by the (weak) 30A-GAL4 induces JAK/STAT activity in the expected expression domain but only where MMP-1 expression and thus JNK/AP1 signaling is low. Conversely, JAK/STAT activ [file pbio.3001665.s003.pdf]

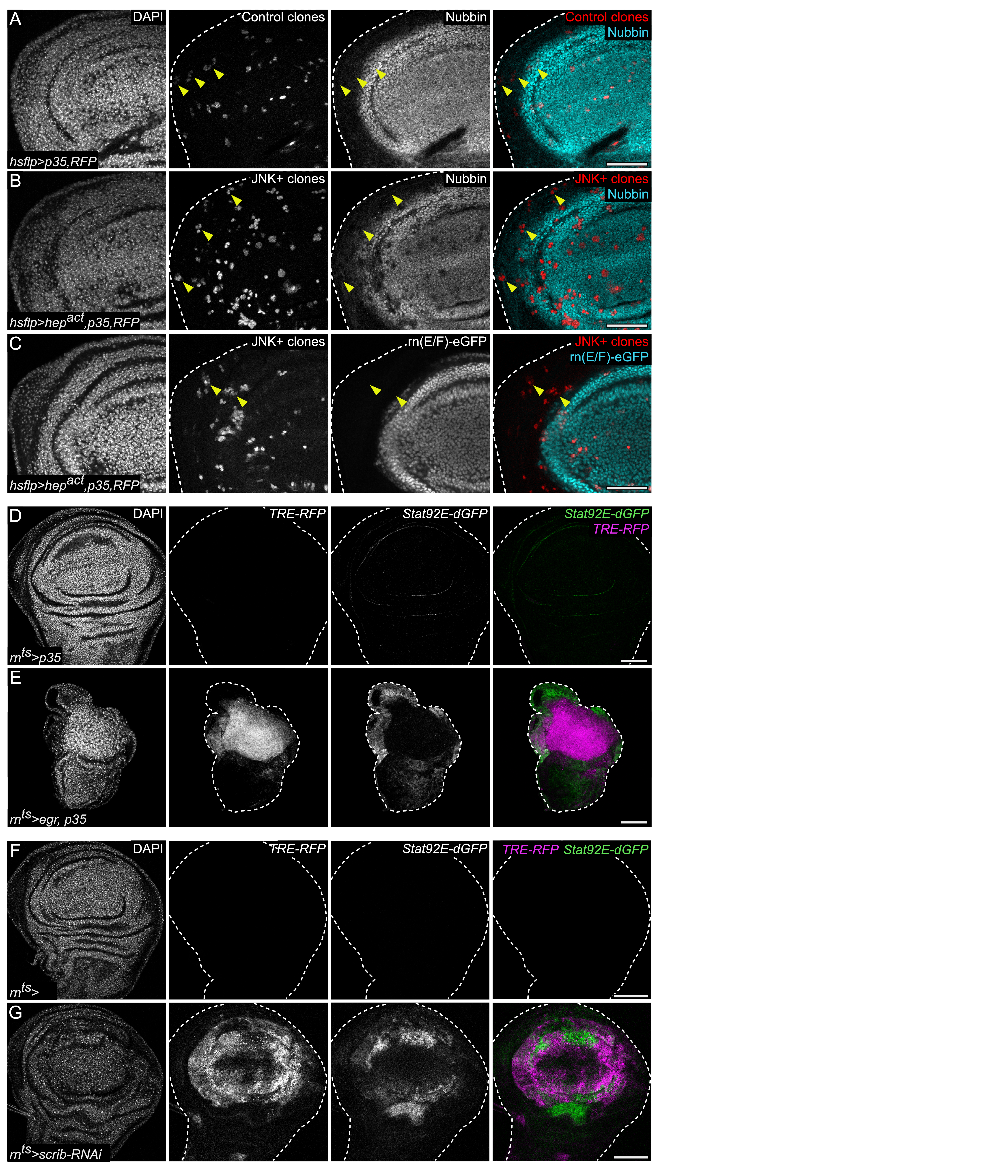

Supplement: S4 Fig — (A, B) p35-expressing control clones (red, A) and p35,hepact-coexpressing clones (JNK+, red, B) at 28 h after clone induction, stained for the pouch-specific transcription factor Nubbin (cyan). JNK/AP-1 signaling clones show no ectopic expression of Nubbin in hinge (yellow arrowheads); however, Nubbin is repressed in clones within the pouch. A total of n = 6, control and n = 6 discs with clones coexpressing p35,hepact were evaluated. (C) p35,hepact-coexpressing clones (red) in a disc expressing the Rn(E/F)-eGFP reporter (cyan) as a readout for the expression of the pouch-specific transcription factor Rotund (Rn) at 28 h after clone induction. JNK/AP-1 signaling clones show no ectopic clonal expression of Rn in the hinge domain (yellow arrowheads). A total of n = 10 discs were evaluated. (D, E) A control (D) and egr,p35-coexpressing disc (E) after 24 h of expression. Discs also express TRE-RFP (magenta) and Stat92E-dGFP (green) reporters. Note the absence of JAK/STAT reporter activity in the central (undead) JNK/AP-1 signaling cells. A total of n = 17, control discs and n = 29, egr,p35-coexpressing discs were evaluated from N = 2 independent experiments. (F, G) A control (F) and scrib-RNAi-coexpressing disc (G) after 44 h of expression in the central rn-GAL4 domain. Discs are also expressing the TRE-RFP (magenta) and Stat92E-dGFP (green) reporters. Note how JNK/AP-1 and JAK/STAT signaling cells in the targeted rn-GAL4 domain largely separate into distinct areas. A total of n = 10, control were evaluated from N = 2 independent experiments. A total of n = 21, scrib-RNAi-coexpressing discs were evaluated from N = 3 independent experiments. Discs were stained with DAPI to visualize nuclei. Scale bars: 50 μm. (TIFF) [file pbio.3001665.s004.tiff]

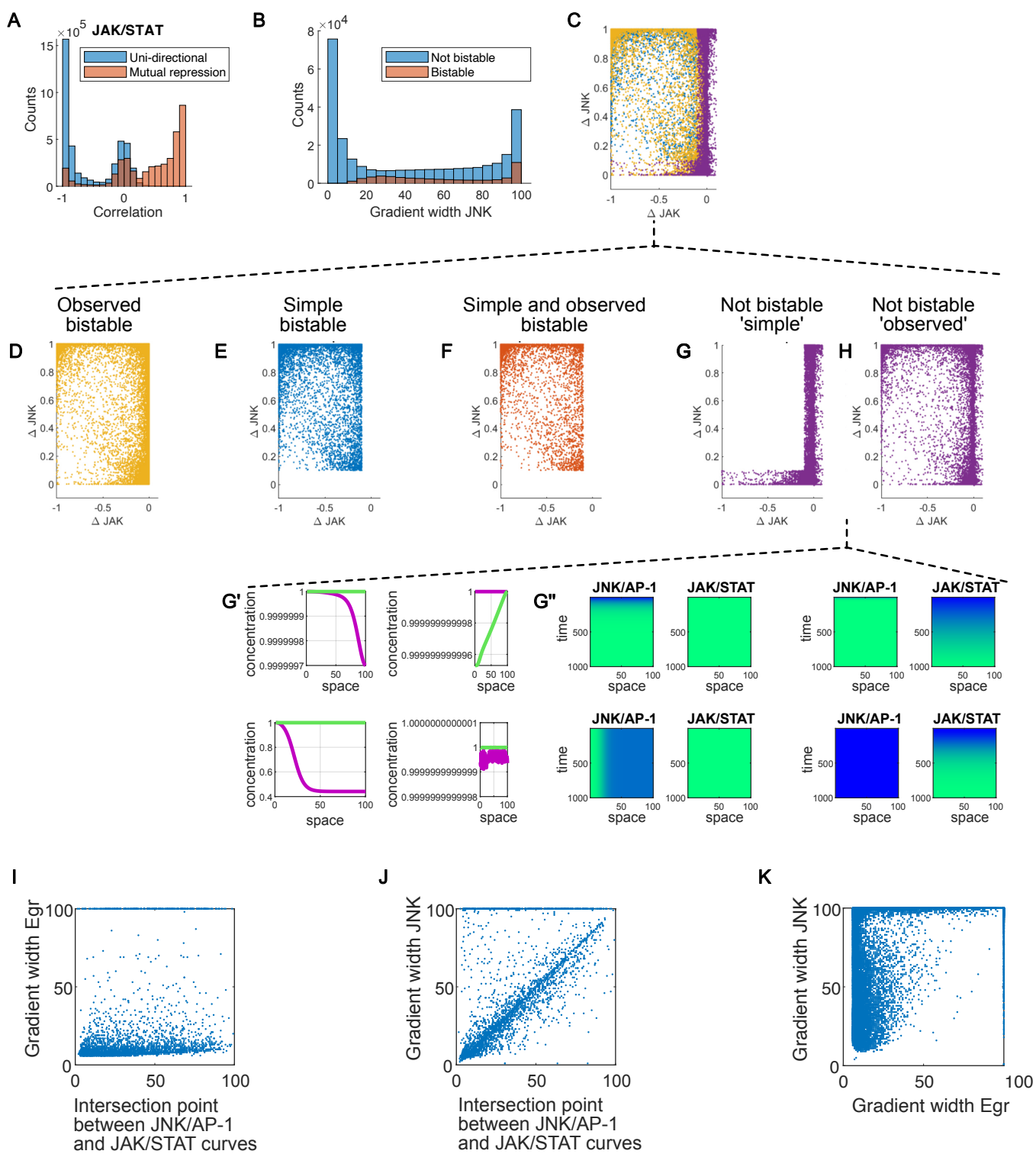

L

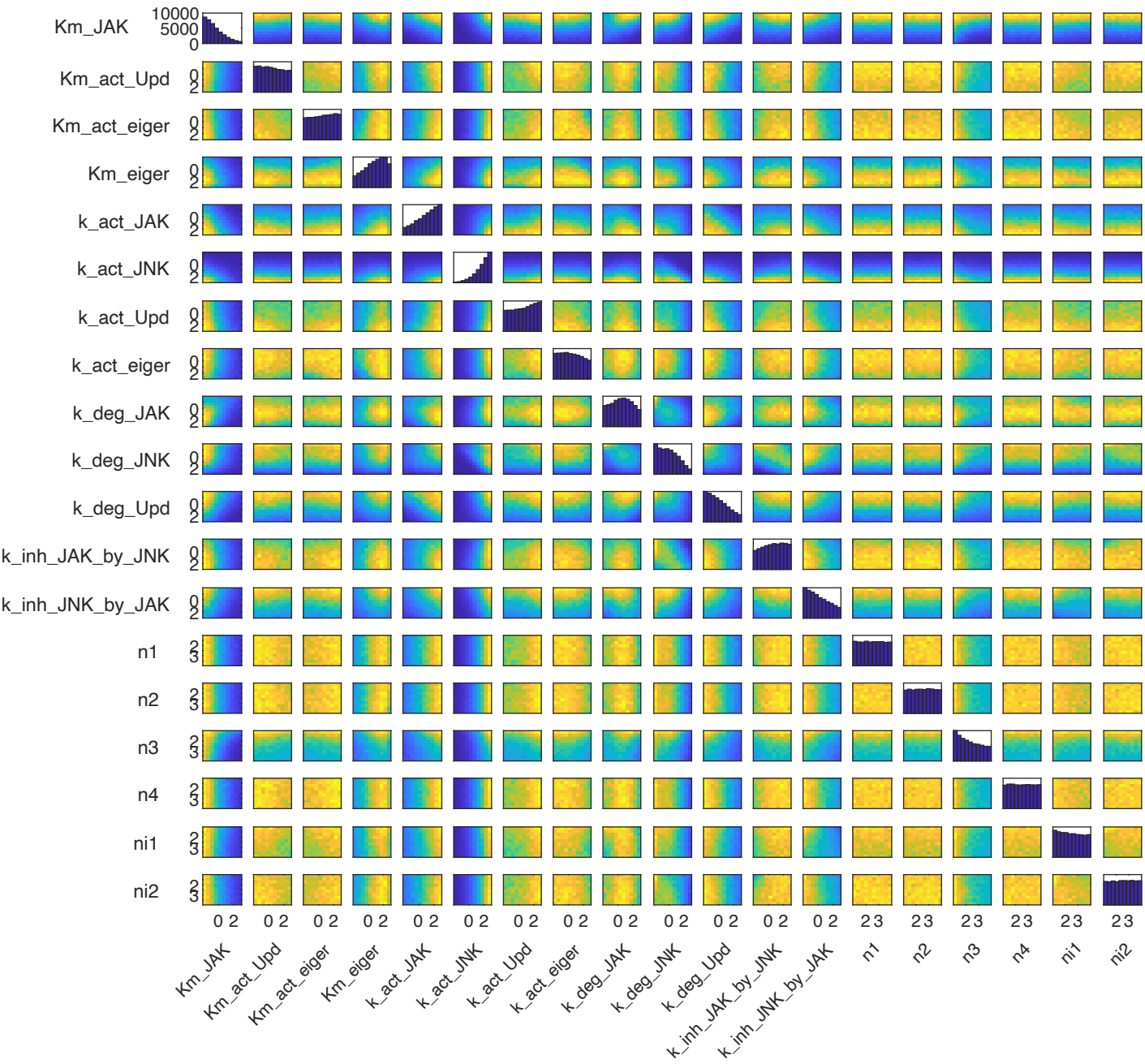

Supplement: S5 Fig — (A) Counts of the correlation coefficients (binned) between the experimental curves of the JAK/STAT gradient shown in Fig 5B and the solutions obtained from the simulation for all simulated parameter sets in the unidirectional (blue) and the mutual repression (red) model. The mutual repression model results in substantially more solutions with positive correlations for the experimentally observed JAK/STAT gradient than the unidirectional repression model. Thus, the mutual repression model is better at recapitulating the response of JAK/STAT to the activation of JNK/AP-1. (B) Counts of “observed bistable” (red) and “not observed bistable” (blue) solutions derived from the mutual repression model examined by determining correlation coefficients between the experimentally and simulation-derived gradient curves (“observed” approach). Counts were plotted against the width of the JNK gradient (spatial position of half of maximum JNK activity) in the simulated steady-state field. No bistable solutions can be found when the JNK-gradient is very narrow, i.e., the correlation coefficients of these solutions with the experimental data is low. (C) Distribution of “bistable” and “not bistable” results obtained by examining the simulated solutions of the mutual repression model using “observed” (correlation coefficient-based) and “simple” (descriptive) criteria. Distribution of all simulated solutions is plotted depending on their differences between minimum and maximum JAK/STAT and JNK activity at steady state in the spatial field. Each point represents a simulation result for a different parameter set. Color code as in (D-H). (D) Subsets of the simulation results shown in (C) classified as “observed bistable.” (E) Subset of the simulation results shown in (C) classified as “simple bistable.” (F) Subset of the simulation results shown in (C) classified as fulfilling both “simple and observed” criteria of bistability. (G) Subset of the simulation results shown in (C) classified a [file pbio.3001665.s005.pdf]

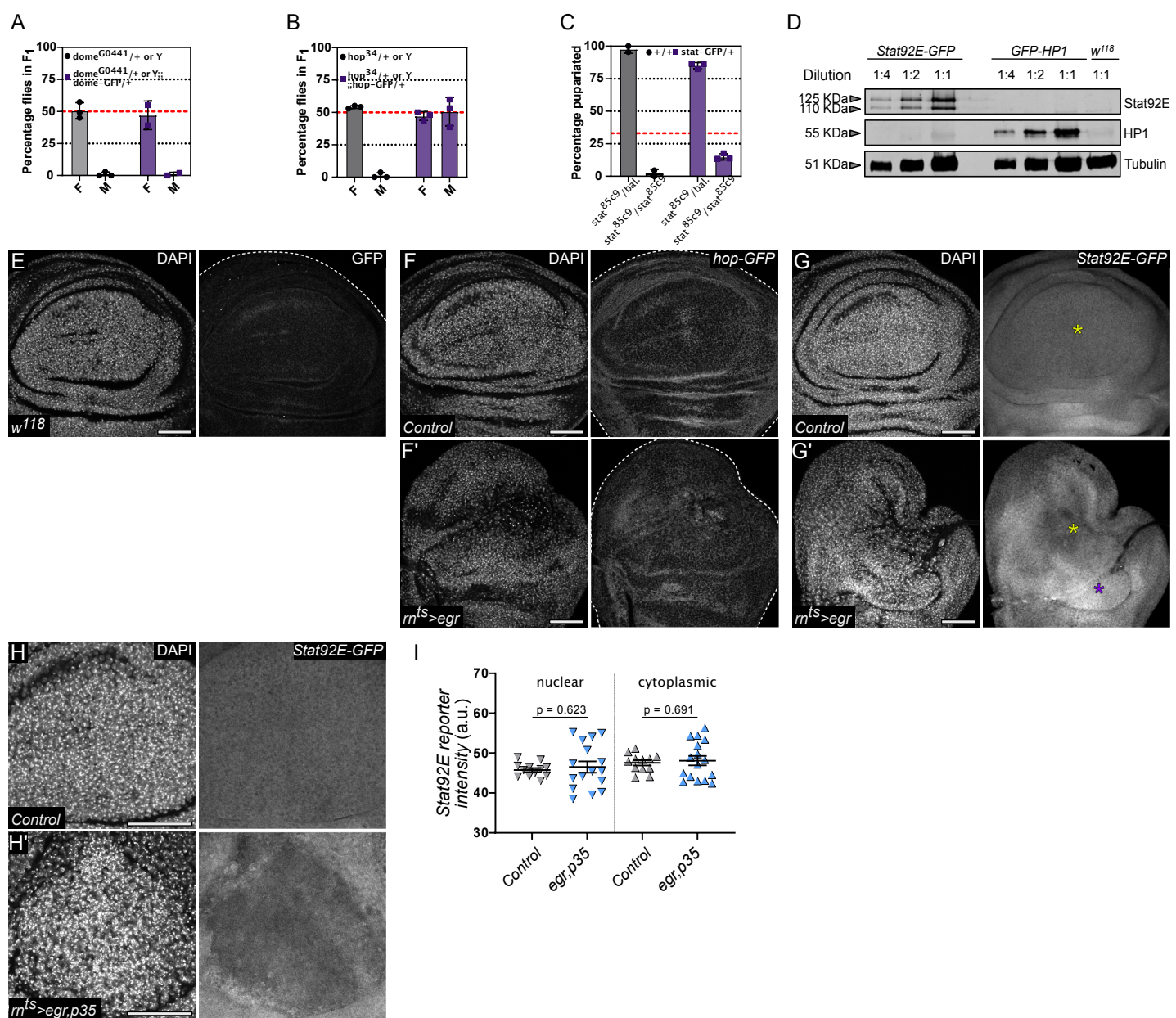

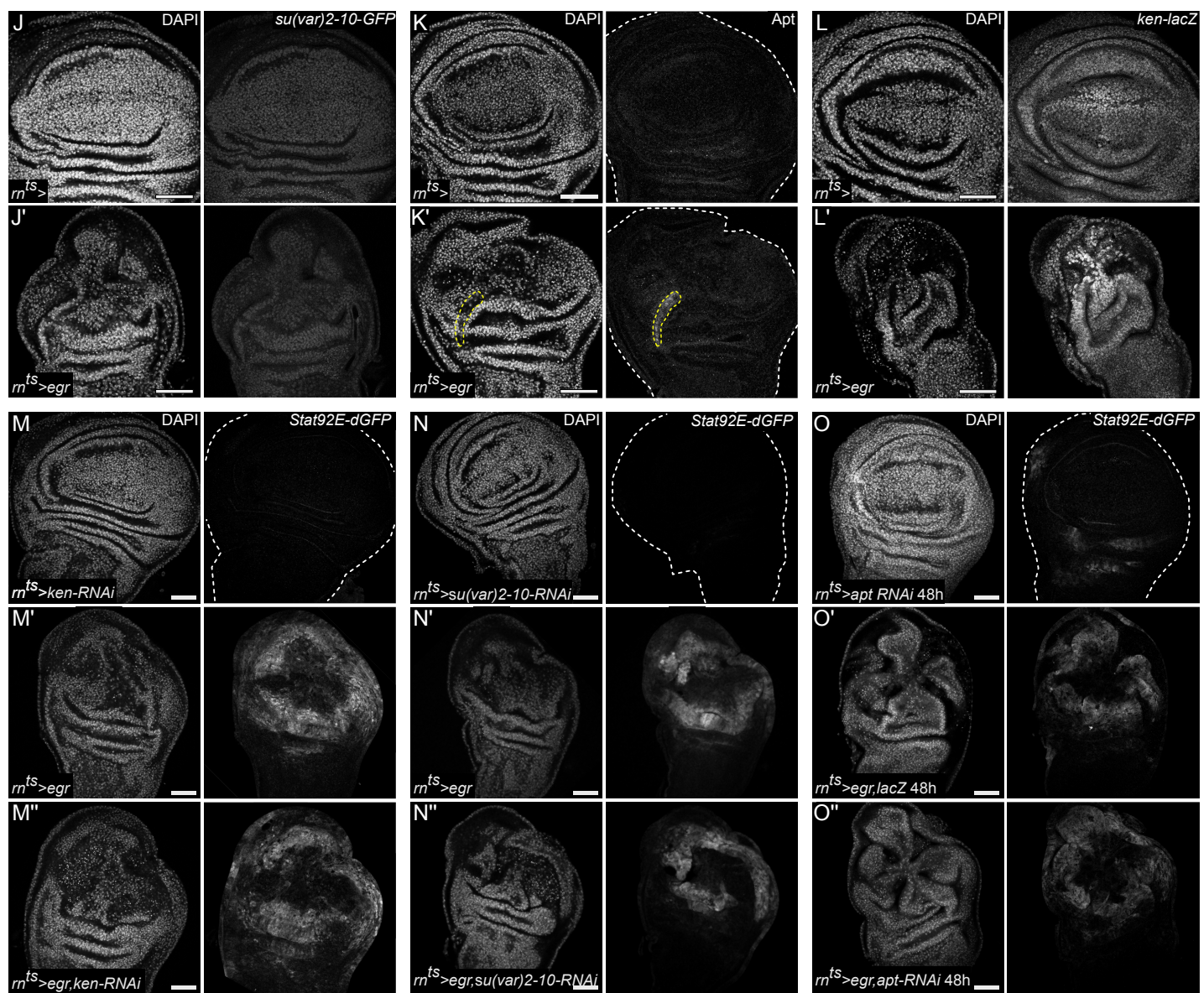

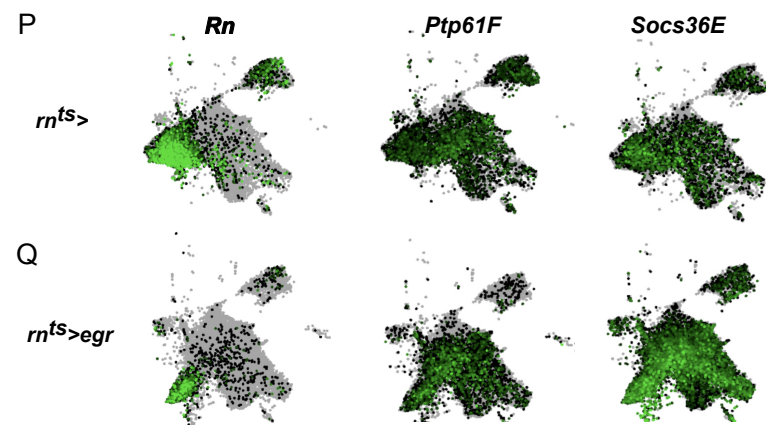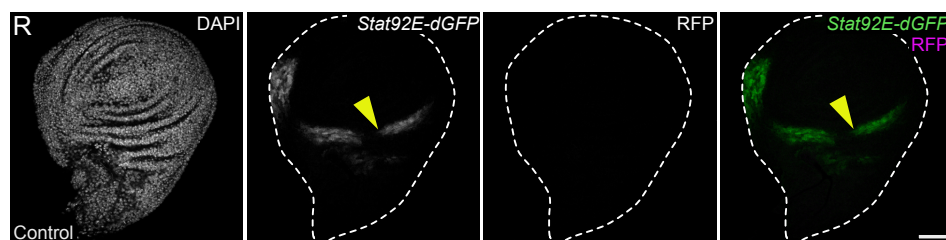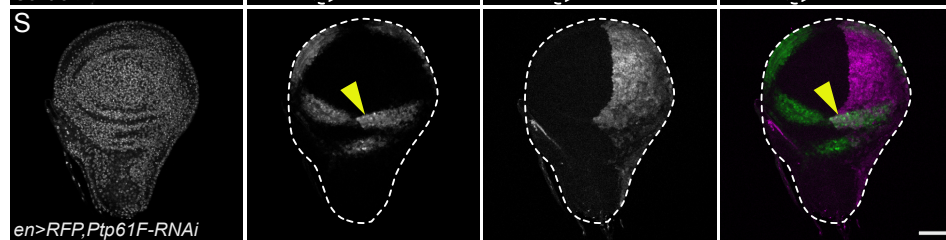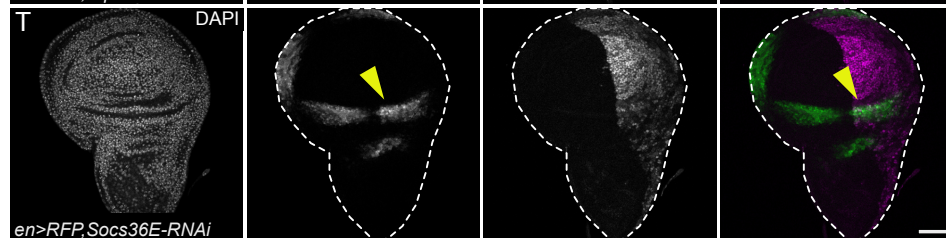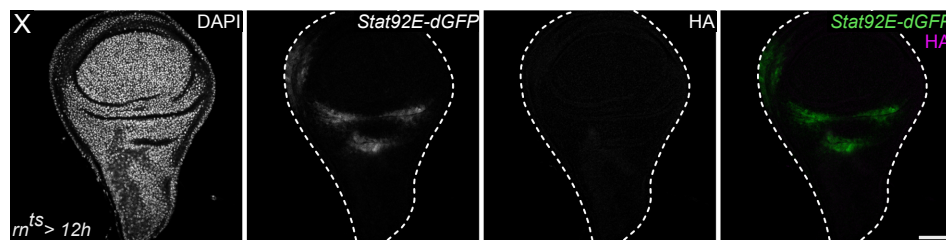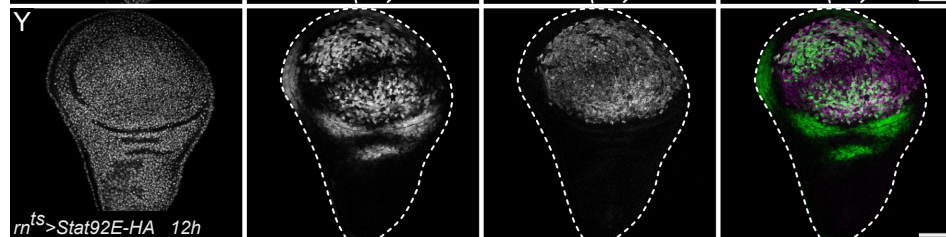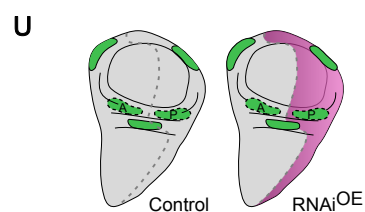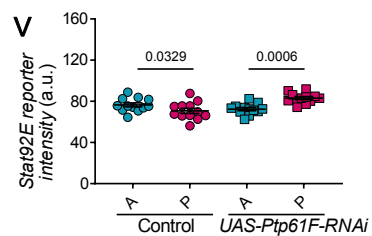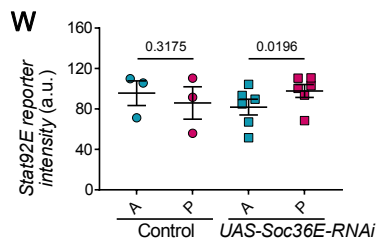

Supplement: S6 Fig — (A-C) Test for genetic complementation of the LOF mutants domeG0441, hop9p5, and stat85C9 (grey bars) by GFP-tagged Dome, Hop, and Stat92E fosmid lines (purple bars) in adult female (F) and male (M) flies (see Experimental procedures). Dome-GFP is unable to rescue male lethality in domeG0441 LOF background to the expected rate of rescue, i.e., 50% of adults in F1 (red dashed line), n = 2 independent replicates (A). Hop-GFP successfully rescues male lethality in hop9p5 LOF background from 0% to 47% of viable adults, at the expected rate of rescue in F1 (red dashed line), n = 3 independent replicates (B). Stat92E-GFP partially rescues larval lethality in stat85c9 null mutants from 0% to 15% pupariation, at approximately half the expected rate of a full rescue (red dashed line), n = 3 independent replicates (C). Thus, the Hop-GFP and Stat92E-GFP fosmid lines were used for further analysis of protein localization in egr-expressing discs. (D) Western blots analyzed for Stat92E-GFP fusion protein expression in imaginal discs. HP-1-GFP genotypes were included as positive control, along with a non-GFP-expressing negative control from wild-type imaginal disc extracts. Increasing concentrations were loaded and probed with anti-GFP and anti-Tubulin antibody. The GFP-tagged Stat92E protein isoforms are detected at the expected MW (125 Kda and 110 Kda) and likely representing 4 overlapping isoforms running in 2 separate weight ranges (71.2–76.8 kD and 85.6–92.8 kD plus GFP-tag). (E) A control w118 disc used as a negative control to determine anti-GFP antibody background. (F, F’) A control (F) and egr-expressing disc (F’) after 24 h of expression also ubiquitously expressing a GFP-tagged Hop protein. A total of n = 5 control and n = 4 egr-expressing discs were evaluated from N = 2 independent experiments. (G, G’) A control (G) and egr-expressing disc (G’) after 24 h of expression also ubiquitously expressing the GFP-tagged Stat92E protein. While Stat92E expression is low in the m [file pbio.3001665.s006.pdf]

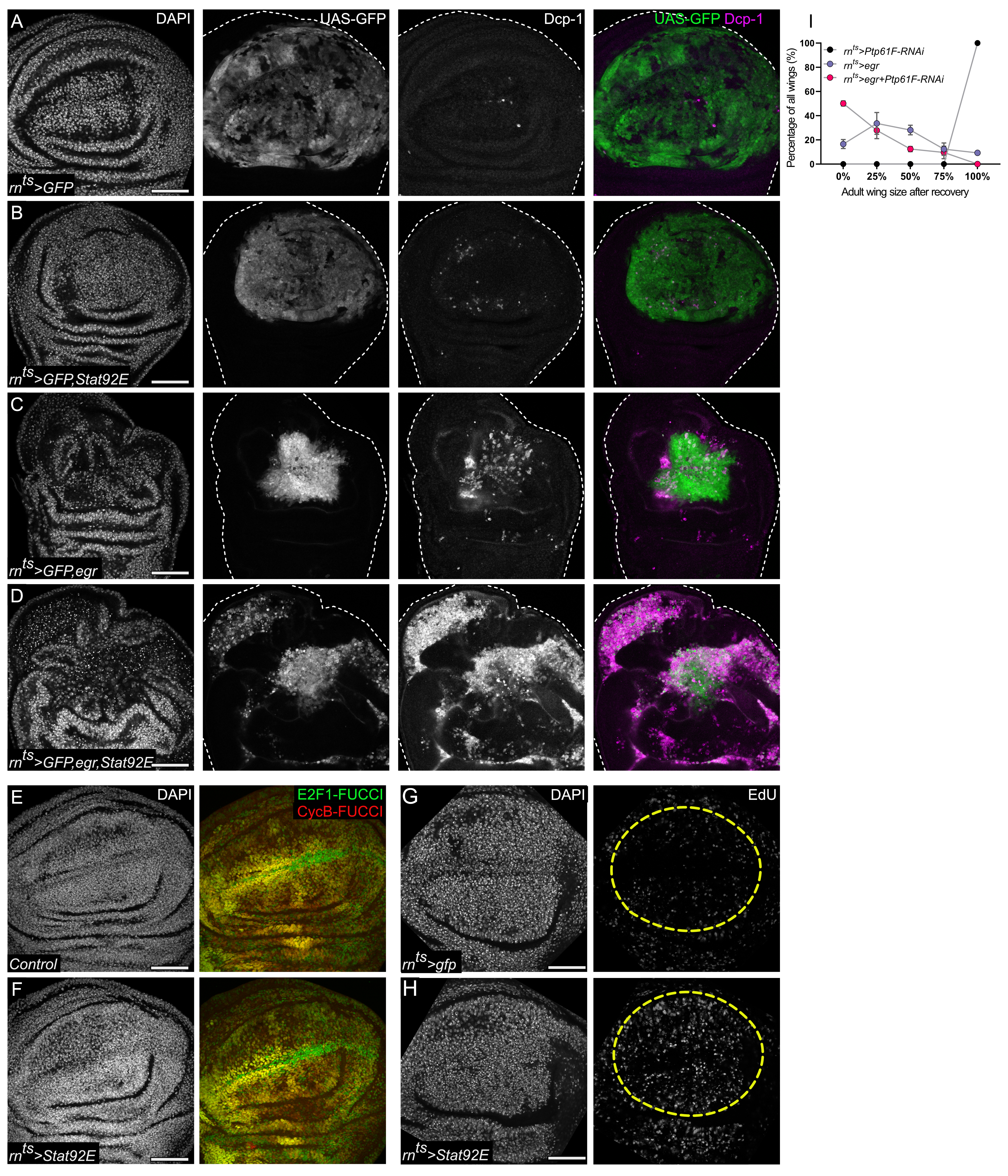

Supplement: S7 Fig — Coactivation of JNK/AP-1 and JAK/STAT signaling causes escape from G2 arrest and apoptosis (A-D). A control wing disc (A), Stat92E-expressing (B), egr-expressing (C), and egr,Stat92E-coexpressing disc (D), which also express UAS-GFP (green), all under the control of rn-GAL4. Discs were stained for cleaved Dcp-1 (magenta) to visualize apoptosis. Note how almost all apoptotic debris is labelled by GFP (D), demonstrating that apoptotic cells truly originate from egr,Stat92E-coexpressing cells. A total of n = 9, control discs; n = 10, Stat92E-expressing discs; n = 11, egr-expressing discs; and n = 18, egr,Stat92E-coexpressing discs were evaluated from N = 2 independent experiments. (E, F) A control (E) and Stat92E-expressing wing disc (F) at R0, also expressing the FUCCI reporter. A total of n = 20, control discs were evaluated from N = 4 independent experiments. A total of n = 17, Stat92E-expressing discs were evaluated from N = 2 independent experiments. (G, H) A gfp-expressing control (G) and Stat92E-expressing disc (H) at R0, assayed for S-phase activity by EdU incorporation. Elevated EdU is detected in the pouch domain ectopically expressing Stat92E. However, no changes were observed in the FUCCI cell cycle profile. However, no changes were observed in the FUCCI cell cycle profile. A total of n = 10, control discs and n = 10, Stat92E-expressing discs were evaluated. (I) Adult wings developing from of UAS-Ptp61F-RNAi expressing; egr-expressing and egr,Ptp61F-RNAi-coexpressing discs after 24 h of expression were scored according to wing size and morphology, as previously described [29,50]. Graphs display mean of binned wing scores emerging from n = 44, Ptp61F-RNAi-expressing; n = 260 egr-expressing and n = 117, egr,Ptp61F-RNAi-expressing discs from N = 4 independent experiments. Source data for quantifications provided in S1 File. Maximum projections of multiple confocal sections are shown in E and F. Discs were stained with DAPI to visualize nuclei. Scale bars: 50 μ [file pbio.3001665.s007.tiff]

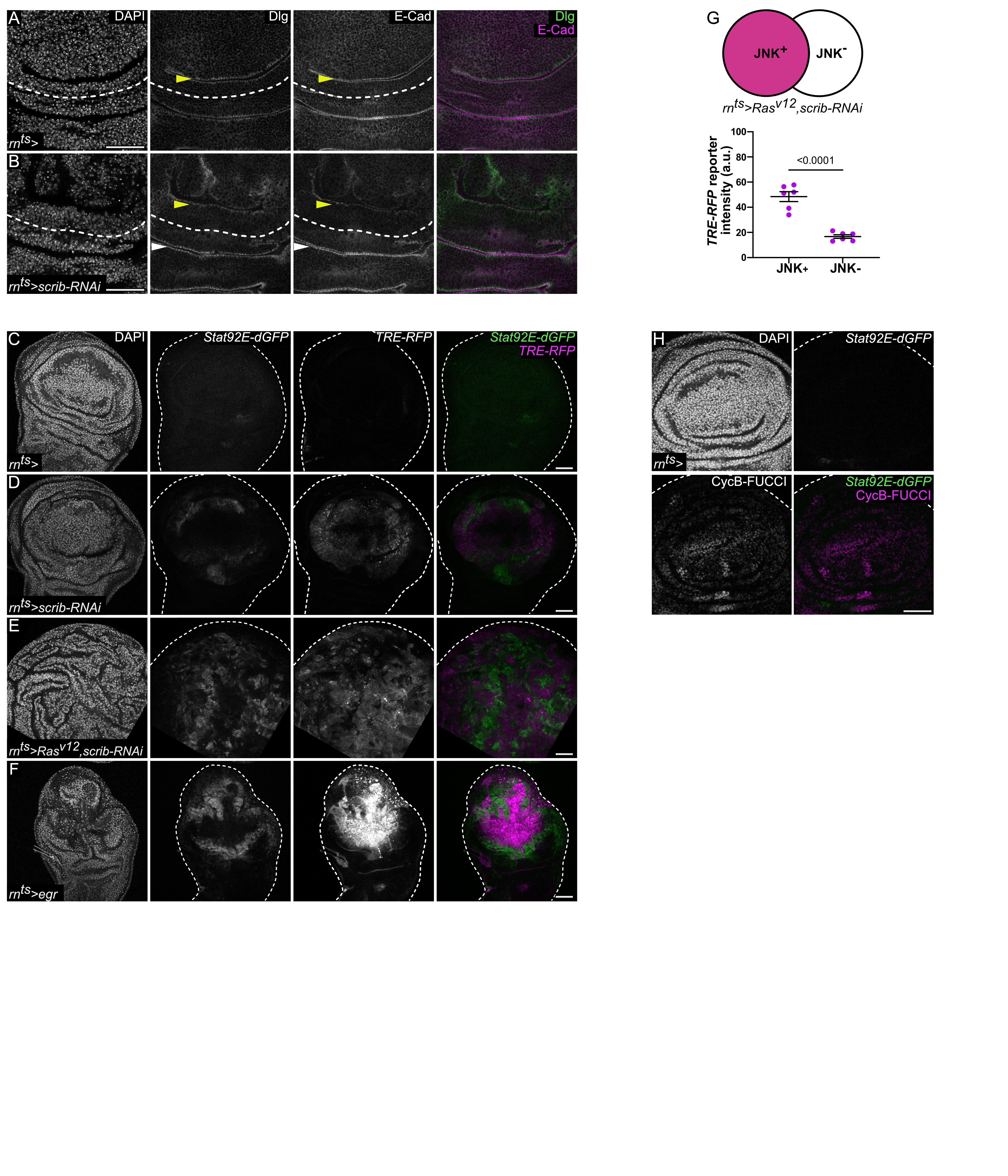

Supplement: S8 Fig — (A, B) A control wing disc (A) and wing discs expressing scrib-RNAi (B) for 44 h stained for E-cadherin (Ecad, adherens junction marker) (magenta) and Discs-large (Dlg, marker of basal polarity) (green) reveals loss of cell adhesion and cell polarity, and thus loss of barrier integrity (yellow arrowheads in B) in the scrib-RNAi-expressing pouch domain when compared to its respective hinge control domain (white arrowheads in B), as well as to the undamaged control pouch (yellow arrowhead in A). The pouch-hinge interface is marked with white dashed lines. A total of n = 3, control and n = 3 scrib-RNAi-expressing discs were evaluated. (C-F) A control wing disc (C) and wing discs expressing scrib-RNAi (D), Rasv12,scrib-RNAi (E), and egr (F) for 44 h. Discs also express the JNK/AP-1 reporter TRE-RFP (magenta) and JAK/STAT reporter Stat92E-dGFP (green). TRE-RFP and Stat92E-dGFP reporter fluorescence intensities were adjusted to subsaturation in egr-expressing discs and all genotypes were imaged at comparable settings; however, brightness settings were raised for all samples equally to prepare the figure. A total of n = 5, control; n = 4, scrib-RNAi-expressing; n = 6, Rasv12,scrib-RNAi-expressing; and n = 7 egr-expressing discs were evaluated. (G) Schematic illustrates TRE-RFP-positive (JNK+, magenta) and TRE-RFP-negative (JNK−, white) regions segmented from images for measurements. Graph represents TRE-RFP reporter fluorescence intensity measured within selected JNK+ and JNK− image masks. Graphs represent mean ± SEM for n = 6, Rasv12,scrib-RNAi-expressing discs. Paired t test was performed to test for statistical significance. (H) A control disc expressing the Stat92E-dGFP (green) reporter and G2-specific FUCCI reporter ubi-mRFP-NLS-CycB1-266 (magenta). A total of n = 6, control discs were evaluated. Source data for quantifications provided in S1 File. Discs were stained with DAPI to visualize nuclei. Scale bars: 50 μm. (TIFF) [file pbio.3001665.s008.tiff]
