# Supplementary material for: Mutual repression between JNK/AP-1 and JAK/STAT stratifies senescent and proliferative cell behaviors during tissue regeneration
Source: PLoS Biol. 2023 May 30;21(5):e3001665. doi: 10.1371/journal.pbio.3001665 (PMC10228795; doi:10.1371/journal.pbio.3001665)
Supplement: S1 Table — Reagents and genetic lines used in this study. (DOCX) [file pbio.3001665.s009.docx]

**Table S1 Key Resource Tables**

| **Designation - Genetic reagent**  **(*D. melanogaster*)** | **Source/Ref.** | **Identifiers** | **Additional information** |
| --- | --- | --- | --- |
| *‘RFP’: Act5C>FRT.CD2>GAL4, UAS-RFP* | PMID: 29494583 | BDSC: 30558 |  |
| *‘apt-RNAi’: UAS-apt-RNAi^TRiP.JF02134^* | PMID: 26320097 | BDSC: 26236 |  |
| *'CycB-FUCCI': Ubi-mRFP1.NLS.CycB^1–266^* | PMID: 24726363 |  |  |
| *‘Dif-GFP’: Pbac(Dif-GFP.FPTB)^VK00033^* |  | BDSC: 42673 |  |
| *'Df(3L)H99': Df(3L)H99, kni[ri-1] p[p]* |  | BDSC: 1576 |  |
| *dome^G0441^* | PMID: 11751581 | BDSC: 12030 |  |
| *‘dome-GFP’: FlyFos (dome::TGVBF)* | PMID: 26896675 | VDRC: 318098 |  |
| *‘E2F1-FUCCI, CycB-FUCCI’; Ubi-GFP.E2f1^1-230^, Ubi-mRFP1.NLS.CycB^1-266^* | PMID: 24726363 | BDSC: 55123 |  |
| *‘egr^ts^’: tub-GAL80ts, UAS-egr* |  |  | I.Hariharan, University of California, Berkeley |
| *‘en>RFP’: enGAL4, UAS-mRFP.NLS* |  | BDSC: 30557 |  |
| *‘G-TRACE’: UAS-FLP.Exel, Ubi-p63E(FRT.STOP)Stinger’* |  | BDSC: 28282 |  |
| *GFP-HP-1* | PMID: 18316477 | BDSC: 30561 |  |
| *‘GFP’: UAS-GFP^S56T^* |  | BDSC: 1521 |  |
| *gstD-GFP* | PMID: 18194654 |  | D. Bohmann, University of Rochester Medical Center |
| *‘hep^act^’: UAS-hep^act^* | PMID: 10903185 | BDSC: 9306 |  |
| *hop^34^* | PMID: 3095163 |  | N. Perrimon |
| *‘hop-GFP’: FlyFos (hop::TGVBF)* | PMID: 26896675 | VDRC: 318158 |  |
| *hsflp^122^* |  |  | D. Bilder, University of California, Berkeley |
| *‘ken-LacZ’: P(PZ)ken^02970^* | PMID: 10471706 | BDSC: 11244 |  |
| *‘ken-RNAi’: UAS-ken-RNAi^TRiP.HMS01219^* | PMID: 26320097 |  |  |
| *‘Ptp61F-RNAi’: UAS-Ptp61F-RNAi ^i2-5^* | PMID: 19398577 | BDSC: 56510 |  |
| *‘p35’': UAS-p35.H* | PMID: 9144202 | BDSC: 5072 |  |
| *rn>GFP':* |  |  |  |
| *‘rn(ts)>’: rn^GAL4^*^-DeltaS^*, tubGAL80^ts^* |  | BDSC: 8142 | recombinant |
| *‘rn(ts)>egr’: rn^GAL4-5^, UAS-egr, tubP-GAL80^ts^* |  | BDSC: 8142; BDSC: 7018 | recombinant; I.Hariharan, University of California, Berkeley |
| *‘Ras^V12^, scrib-RNAi’: UAS-Ras^V12^,scrib-RNAi* |  |  | M. Uhlirova, University of Cologne |
| *‘Ras^V12^’: UAS-Ras^V12^* |  |  | H. Richardson, University of Melbourne |
| *‘rnE/F-EGFP’: rn(E/F)-EGFP (ds Red+)* | PMID: 26497147 |  | P. Volkan, Duke University School of Medicine |
| *‘scrib-RNAi': UAS-scrib-RNAi* |  |  | M. Uhlirova, University of Cologne |
| *‘Socs36E-RNAi’: UAS-Socs36e-RNAi* |  | BDSC: 35036 |  |
| *‘Stat92E’: UAS-Stat92E.ORF.3xHA* | PMID: 23583758 | Fly-ORF F000750 |  |
| *‘Stat92E^85c9^ ’: FRT82B stat92E ^85c9^* |  |  | E. Bach, New York University School of Medicine |
| *‘Stat92E-dGFP’: 10xStat92E-dGFP* | PMID: 17008134 |  |  |
| *‘Stat92E-GFP’: 10xStat92E-GFP* | PMID: 17008134 |  |  |
| *‘Stat92E-GFP’: PBac(Stat92E-GFP.FLAG)^VK00037^* |  | BDSC: 38670 |  |
| *‘Su(var)2-10-GFP’: PBac(Su(var)2-10-GFP.FPTB)^VK00037^* | PMID: 29284660 | BDSC: 64795 |  |
| *‘Su(var)2-10-RNAi’: UAS- Su(var)2-10-RNAi ^TRiP.HMS00750^* | PMID: 26320097 | BDSC: 32956 |  |
| *‘TRE-RFP’: TRE-DsRed.T4* | PMID: 22509270 |  |  |
| *‘Xbp1-GFP’: UAS-Xbp1-GFP.HG* | PMID: 23160805 | BDSC: 60731 |  |
| *upd3.1-3-LacZ* |  |  | I.Hariharan, University of California, Berkeley |
| *upd-LacZ: upd>LacZ (PD1)* | PMID: 8582614 |  | Y. H. Sun, Academia Sinica, Taipei |
| *w^118^* |  |  |  |
| *‘30A>': 30AGAL4* |  | BDSC: 37534 |  |

| **Antibodies / Commercial Kits** | **Source/Reference** | **Identifiers** | **Dilution** |
| --- | --- | --- | --- |
| Chicken anti-GFP | Abcam | Cat. #: ab13970 | (1:1000) |
| Mouse anti-H3-pS10 | Abcam | Cat. #: ab14955 | (1:2000) |
| Mouse anti-β-Galactosidase | Promega | Cat. #: Z3782 | (1:1000) |
| Mouse monoclonal anti-MMP-1 | DSHB | Cat. #: 3A6B4 | (1:30) |
| Mouse monoclonal anti-MMP-1 | DSHB | Cat. #: 3B8D12 | (1:30) |
| Mouse monoclonal anti-MMP-1 | DSHB | Cat. #: 5H7B11 | (1:30) |
| Mouse monoclonal anti-Nubbin | DSHB | Cat. #: 2D4 | (1:100) |
| Mouse monoclonal anti-α-Tubulin | Sigma-Aldrich | Cat. #: T9026 | (1:5000) |
| Rabbit anti-cleaved Dcp-1 | Cell Signaling | Cat. #: 9578 | (1:200) |
| Rabbit monoclonal anti-GFP | Invitrogen | Cat. #: G10362 | (1:200) |
| Rabbit polyclonal anti-apt | R. Schuh, MPI-MS, Göttingen | NA | (1:200) |
| Rat monoclonal anti-GFP | Chromotek | Cat. #: 3H9-100 | (1:1000) |
| Rat monoclonal anti-HA | Monoclonal Antibody Core Facillity Helmholtz Zentrum München | Clone #: 3F10 | (1:20) |
| Rat monoclonal anti-RFP |  | Clone #: 5F8 | (1:20) |
| Phalloidin-TRITC | Sigma Aldrich | Cat. #: P1951 | (1:500) |
| Goat anti-chicken IgY Alexa Flour 488 | Invitrogen | Cat. #: A-11039 | (1:500) |
| Goat anti-mouse IgG Alexa Flour 488 | Invitrogen | Cat. #: A-11001 | (1:500) |
| Goat anti-rabbit IgG Alexa Flour 488 | Invitrogen | Cat. #: A-11008 | (1:500) |
| Goat anti-rat IgG Alexa Flour 488 | Invitrogen | Cat. #: A-11006 | (1:500) |
| Goat anti-rat IgG Alexa Flour 488 pre-adsorbed | Abcam | Cat. #: ab150165 | (1:500) |
| Goat anti-mouse IgG Alexa Flour 555 | Invitrogen | Cat. #: A-21422 | (1:500) |
| Donkey anti-mouse IgG Alexa Flour 555 pre-adsorbed | Abcam | Cat. #: ab150110 | (1:500) |
| Goat anti-rat IgG Alexa Flour 555 | Invitrogen | Cat. #: A-21434 | (1:500) |
| Donkey anti-rat IgG Alexa Flour 555 | Abcam | Cat. #: ab150154 | (1:500) |
| Goat anti-mouse Alexa Flour 647 | Invitrogen | Cat. #: A-21235 | (1:500) |
| Donkey anti-mouse Alexa Flour 647 pre-adsorbed | Abcam | Cat. #: ab150111 | (1:500) |
| Goat anti-rat Alexa Flour 647 | Invitrogen | Cat. #: A-21247 | (1:500) |
| Goat anti-rabbit Alexa Flour 647 | Invitrogen | Cat. #: A-21244 | (1:500) |
| Click-iT Plus EdU Alexa Fluor 647 Imaging Kit | Invitrogen | Cat. #: C10640 |  |
| CellEvent Senescence Green Detection Kit | Invitrogen | Cat. #: C10850 |  |
| PMID – PubMed Identifier; BDSC – Bloomington Drosophila Stock Center; DSHB – Developmental Studies Hybridoma Bank, VDRC – Vienna Drosophila Stock Collection, FlyORF – University of Zurich ORFeome Project | | | |
