# Supplementary material for: Mutual repression between JNK/AP-1 and JAK/STAT stratifies senescent and proliferative cell behaviors during tissue regeneration
Source: PLoS Biol. 2023 May 30;21(5):e3001665. doi: 10.1371/journal.pbio.3001665 (PMC10228795; doi:10.1371/journal.pbio.3001665)
Supplement: S2 Table — Detailed genotypes listed per figure panel. (DOCX) [file pbio.3001665.s010.docx]

**Table S2. Detailed genotypes**

| **Figure** | **Panel** | **Genotype** | **Temperature Shift / Recovery** |
| --- | --- | --- | --- |
| Fig. 1 | A | UAS-GFP; rn-GAL4 | 18ᵒC, till D7 AED |
| Fig. 1 | C, E, G, I, K, M | TRE-RFP; 10xStat92E-dGFP/rn-GAL4, tub-GAL80(ts) | 7h, 14h or 24h 30ᵒC, D7 AED |
| Fig. 1 | D, F, H, J, L, N | TRE-RFP; 10xStat92E-dGFP/rn-GAL4, tub-GAL80(ts), UAS-egr | 7h, 14h or 24h 30ᵒC, D7 AED |
| Fig. 1 | O, Q, S | TRE-RFP; rn-GAL4, tub-GAL80(ts) | 7h, 14h or 24h 30ᵒC, D7 AED |
| Fig. 1 | P, R, T | TRE-RFP; rn-GAL4, tub-GAL80(ts), UAS-egr | 7h, 14h or 24h 30ᵒC, D7 AED |
| Fig. 1 | U, W, Y | Ubi-GFP.E2f[1-230], Ubi-mRFP1.NLS.CycB[1-266]; rn-GAL4, tub-GAL80(ts) | 7h, 14h or 24h 30ᵒC, D7 AED |
| Fig. 1 | V, X, Z | Ubi-GFP.E2f[1-230], Ubi-mRFP1.NLS.CycB[1-266]; rn-GAL4, tub-GAL80(ts), UAS-egr | 7h, 14h or 24h 30ᵒC, D7 AED |
| Fig. S1 | B | ;;rn-GAL4, tub-GAL80(ts) | 24h 30ᵒC, D7 AED |
| Fig. S1 | C | ;;rn-GAL4, tub-GAL80(ts), UAS-egr | 24h 30ᵒC, D7 AED |
| Fig. S1 | D | TRE-RFP; rn-GAL4, tub-GAL80(ts) | 24h 30ᵒC, D7 AED |
| Fig. S1 | E | TRE-RFP; rn-GAL4, tub-GAL80(ts), UAS-egr | 24h 30ᵒC, D7 AED |
| Fig. S1 | F, H | TRE-RFP; 10xStat92E-dGFP/rn-GAL4, tub-GAL80(ts) | 7h or 14h 30ᵒC, D7 AED |
| Fig. S1 | G, I | TRE-RFP; 10xStat92E-dGFP/rn-GAL4, tub-GAL80(ts), UAS-egr | 7h or 14h 30ᵒC, D7 AED |
| Fig. 2 | A | TRE-RFP; rn-GAL4, tub-GAL80(ts) | 24h 30ᵒC, D7 AED |
| Fig. 2 | B | TRE-RFP; rn-GAL4, tub-GAL80(ts), UAS-egr | 24h 30ᵒC, D7 AED |
| Fig. 2 | D | TRE-RFP; Upd3.1-3-LacZ/rn-GAL4, tub-GAL80(ts) | 24h 30ᵒC, D7 AED |
| Fig. 2 | E | TRE-RFP; Upd3.1-3-LacZ/rn-GAL4, tub-GAL80(ts), UAS-egr | 24h 30ᵒC, D7 AED |
| Fig. 2 | G | Su(var)2-10 GFP/rn-GAL4, tub-GAL80(ts) | 24h 30ᵒC, D7 AED |
| Fig. 2 | H | Su(var)2-10 GFP/rn-GAL4, tub-GAL80(ts), UAS-egr | 24h 30ᵒC, D7 AED |
| Fig. 2 | J | gstD-GFP; rn-GAL4, tub-GAL80(ts) | 24h 30ᵒC, D7 AED |
| Fig. 2 | K | gstD-GFP; rn-GAL4, tub-GAL80(ts), UAS-egr | 24h 30ᵒC, D7 AED |
| Fig. 2 | M | TRE-RFP/Dif-GFP; rn-GAL4, tub-GAL80(ts) | 24h 30ᵒC, D7 AED |
| Fig. 2 | N | TRE-RFP/Dif-GFP; rn-GAL4, tub-GAL80(ts), UAS-egr | 24h 30ᵒC, D7 AED |
| Fig. 2 | P | UAS-Xbp1-GFP.HG/ rn-GAL4, tub-GAL80(ts) | 24h 30ᵒC, D7 AED |
| Fig. 2 | Q | UAS-Xbp1-GFP.HG/ rn-GAL4, tub-GAL80(ts), UAS-egr | 24h 30ᵒC, D7 AED |
| Fig. S2 | A, C | Upd-LacZ/X; TRE-RFP; TM6B, tub-GAL80 | 24h 30ᵒC, D7 AED |
| Fig. S2 | B, C | Upd-LacZ/X; TRE-RFP; rn-GAL4, tub-GAL80(ts), UAS-egr | 24h 30ᵒC, D7 AED |
| Fig. 3 | A, C | TRE-RFP; Upd3.1-3-LacZ/rn-GAL4, tub-GAL80(ts), UAS-egr | 24h 30ᵒC, D7 AED |
| Fig. 3 | D | TRE-RFP; 10xStat92E-dGFP/rn-GAL4, tub-GAL80(ts) | 24h 30ᵒC, D7 AED |
| Fig. 3 | E-G | TRE-RFP; 10xStat92E-dGFP/rn-GAL4, tub-GAL80(ts), UAS-egr | 24h 30ᵒC, D7 AED |
| Fig. 3 | H | en-GAL4, UAS-RFP; 10xStat92E-dGFP/ tub-GAL80(ts), UAS-egr | 24h 30ᵒC, D7 AED |
| Fig. 3 | I-O | TRE-RFP; 10xStat92E-dGFP/rn-GAL4, tub-GAL80(ts), UAS-egr | 7h, 14h or 24h 30ᵒC, D7 AED |
| Fig. S3 | B-G | TRE-RFP; 10xStat92E-dGFP/rn-GAL4, tub-GAL80(ts) | 0h, 7h or 14h 30ᵒC, D7 AED |
| Fig. S3 | H-M | TRE-RFP; 10xStat92E-dGFP/rn-GAL4, tub-GAL80(ts), UAS-egr | 0h, 7h or 14h 30ᵒC, D7 AED |
| Fig. S3 | N | 10xStat92E-dGFP; | 24h 30ᵒC, D7 AED |
| Fig. S3 | O | 30A-GAL4/10xStat92E-dGFP; tub-GAL80(ts), UAS-egr | 24h 30ᵒC, D7 AED |
| Fig. S3 | P-V | TRE-RFP; 10xStat92E-dGFP/rn-GAL4, tub-GAL80(ts) | 7h, 14h or 24h 30ᵒC, D7 AED |
| Fig. 4 | A, C | hsflp[122]; UAS-p35; 10xStat92E-dGFP/Act5C.GAL4 (FRT.CD2), UAS-RFP | 10' HS 37ᵒC, D7 AED, R28h |
| Fig. 4 | B, D | hsflp[122]; UAS-p35/UAS-hep[act]; 10xStat92E-dGFP/Act5C.GAL4 (FRT.CD2), UAS-RFP | 10' HS 37ᵒC, D7 AED, R28h |
| Fig. 4 | E | hsflp[122]; UAS-p35/UAS-hep[act]; 10xStat92E-dGFP/Act5C.GAL4 (FRT.CD2), UAS-RFP | 7' HS 37ᵒC, D5 AED, R48h |
| Fig. 4 | F | TRE-RFP/10xStat92E-dGFP; rn-GAL4, tub-GAL80(ts)/Df(3L)H99 | 24h 30ᵒC, D7 AED |
| Fig. 4 | G, I, K | TRE-RFP/10xStat92E-dGFP; rn-GAL4, tub-GAL80(ts), UAS-egr/Df(3L)H99 | 24h 30ᵒC, D7 AED |
| Fig. 4 | H, K | TRE-RFP/10xStat92E-dGFP; rn-GAL4, tub-GAL80(ts), UAS-egr | 24h 30ᵒC, D7 AED |
| Fig. 4 | L, M | UAS-p35/10xStat92E-dGFP; UAS-scrib RNAi/rn-GAL4, tub-GAL80(ts) | 44h 30ᵒC, D6 AED |
| Fig. S4 | A | hsflp[122]; UAS-p35; 10xStat92E-dGFP/Act5C.GAL4 (FRT.CD2), UAS-RFP | 7' HS 37ᵒC, D7 AED, R28h |
| Fig. S4 | B | hsflp[122]; UAS-p35/UAS-hep[act]; 10xStat92E-dGFP/Act5C.GAL4 (FRT.CD2), UAS-RFP | 7' HS 37ᵒC, D7 AED, R28h |
| Fig. S4 | C | hsflp[122]; UAS-p35; rn(E/F)-EGFP/Act5C.GAL4 (FRT.CD2), UAS-RFP | 7' HS 37ᵒC, D7 AED, R28h |
| Fig. S4 | D | UAS-p35/TRE-RFP; 10xStat92E-dGFP/rn-GAL4, tub-GAL80(ts) | 24h 30ᵒC, D7 AED |
| Fig. S4 | E | UAS-p35/TRE-RFP; 10xStat92E-dGFP/rn-GAL4, tub-GAL80(ts), UAS-egr | 24h 30ᵒC, D7 AED |
| Fig. S4 | F | TRE-RFP; 10xStat92E-dGFP/rn-GAL4, tub-GAL80(ts) | 44h 30ᵒC, D6 AED |
| Fig. S4 | G | UAS-scrib RNAi/TRE-RFP; 10xStat92E-dGFP/rn-GAL4, tub-GAL80(ts) | 44h 30ᵒC, D6 AED |
| Fig. 6 | A | 10xStat92E-dGFP/UAS-Ptp61F RNAi (i2-5); rn-GAL4, tub-GAL80(ts) | 24h 30ᵒC, D7 AED |
| Fig. 6 | B, G | 10xStat92E-dGFP; rn-GAL4, tub-GAL80(ts), UAS-egr | 24h 30ᵒC, D7 AED |
| Fig. 6 | C, G | 10xStat92E-dGFP/UAS-Ptp61F RNAi (i2-5); rn-GAL4, tub-GAL80(ts), UAS-egr | 24h 30ᵒC, D7 AED |
| Fig. 6 | D | 10xStat92E-dGFP; rn-GAL4, tub-GAL80(ts)/UAS-Socs36E-RNAi (HMS01450) | 24h 30ᵒC, D7 AED |
| Fig. 6 | E, H | 10xStat92E-dGFP; rn-GAL4, tub-GAL80(ts), UAS-egr | 24h 30ᵒC, D7 AED |
| Fig. 6 | F, H | 10xStat92E-dGFP; rn-GAL4, tub-GAL80(ts), UAS-egr/UAS-Socs36E-RNAi (HMS01450) | 24h 30ᵒC, D7 AED |
| Fig. 6 | I, M | CycE-lacZ/10xStat92E-dGFP; rn-GAL4, tub-GAL80(ts) | 24h 30ᵒC, D7 AED |
| Fig. 6 | J, M | CycE-lacZ/10xStat92E-dGFP; UAS-Stat92E-3xHA/rn-GAL4, tub-GAL80(ts) | 24h 30ᵒC, D7 AED |
| Fig. 6 | K, M | CycE-lacZ/10xStat92E-dGFP; rn-GAL4, tub-GAL80(ts), UAS-egr | 24h 30ᵒC, D7 AED |
| Fig. 6 | L, M | CycE-lacZ/10xStat92E-dGFP; UAS-Stat92E-3xHA/rn-GAL4, tub-GAL80(ts), UAS-egr | 24h 30ᵒC, D7 AED |
| Fig. S6 | A | dome[G0441];; | - |
| Fig. S6 | A | dome[G0441];;dome-GFP | - |
| Fig. S6 | B | Hop[34];; | - |
| Fig. S6 | B | Hop[34];;hop-GFP | - |
| Fig. S6 | C | Stat92E[85c9]/TM6c or stat92E[85c9]/stat92E[85c9] | - |
| Fig. S6 | C | Stat92E-GFP; stat92E[85c9]/TM6c or Stat92E-GFP; stat92E[85c9]/stat92E[85c9] | - |
| Fig. S6 | D | Stat92E-GFP; | - |
| Fig. S6 | D | GFP-HP1 | - |
| Fig. S6 | D | w[118] | - |
| Fig. S6 | E | w[118] | 24h 30ᵒC, D7 AED |
| Fig. S6 | F | ;;hop-GFP/TM6B, tub-GAL80 | 24h 30ᵒC, D7 AED |
| Fig. S6 | F’ | ;;hop-GFP/rn-GAL4, tub-GAL80(ts), UAS-egr | 24h 30ᵒC, D7 AED |
| Fig. S6 | G | Stat92E-GFP; | 24h 30ᵒC, D7 AED |
| Fig. S6 | G’ | Stat92E-GFP; rn-GAL4, tub-GAL80(ts), UAS-egr | 24h 30ᵒC, D7 AED |
| Fig. S6 | H, I | Stat92E-GFP; | 24h 30ᵒC, D7 AED |
| Fig. S6 | H’, I | p35/Stat92E-GFP; rn-GAL4, tub-GAL80(ts), UAS-egr | 24h 30ᵒC, D7 AED |
| Fig. S6 | J | Su(var)2-10-GFP; rn-GAL4, tub-GAL80(ts) | 24h 30ᵒC, D7 AED |
| Fig. S6 | J’ | Su(var)2-10-GFP; rn-GAL4, tub-GAL80(ts), UAS-egr | 24h 30ᵒC, D7 AED |
| Fig. S6 | K | TRE-RFP; 10xStat92E-dGFP/rn-GAL4, tub-GAL80(ts) | 24h 30ᵒC, D7 AED |
| Fig. S6 | K’ | TRE-RFP; 10xStat92E-dGFP/rn-GAL4, tub-GAL80(ts), UAS-egr | 24h 30ᵒC, D7 AED |
| Fig. S6 | L | ken-LacZ[02970]; rn-GAL4, tub-GAL80(ts) | 24h 30ᵒC, D7 AED |
| Fig. S6 | L’ | ken-LacZ[02970]; rn-GAL4, tub-GAL80(ts), UAS-egr | 24h 30ᵒC, D7 AED |
| Fig. S6 | M | 10xStat92E-dGFP/UAS-ken RNAi (TRiP.HMS01219); rn-GAL4, tub-GAL80(ts) | 24h 30ᵒC, D7 AED |
| Fig. S6 | M’ | 10xStat92E-dGFP; rn-GAL4, tub-GAL80(ts), UAS-egr | 24h 30ᵒC, D7 AED |
| Fig. S6 | M’’ | 10xStat92E-dGFP/UAS-ken RNAi (TRiP.HMS01219); rn-GAL4, tub-GAL80(ts), UAS-egr | 24h 30ᵒC, D7 AED |
| Fig. S6 | N | 10xStat92E-dGFP; UAS-Su(var)2-10 RNAi (TRiP.HMS00750)/rn-GAL4, tub-GAL80(ts) | 24h 30ᵒC, D7 AED |
| Fig. S6 | N’ | 10xStat92E-dGFP; rn-GAL4, tub-GAL80(ts), UAS-egr | 24h 30ᵒC, D7 AED |
| Fig. S6 | N’’ | 10xStat92E-dGFP; UAS-Su(var)2-10 RNAi (TRiP.HMS00750)/rn-GAL4, tub-GAL80(ts), UAS-egr | 24h 30ᵒC, D7 AED |
| Fig. S6 | O | 10xStat92E-dGFP; UAS-apt RNAi (TRiP.JF02134)/rn-GAL4, tub-GAL80(ts) | 48h 30ᵒC, D7 AED |
| Fig. S6 | O’ | 10xStat92E-dGFP; rn-GAL4, tub-GAL80(ts), UAS-egr | 48h 30ᵒC, D7 AED |
| Fig. S6 | O’’ | 10xStat92E-dGFP; UAS-apt RNAi (TRiP.JF02134)/rn-GAL4, tub-GAL80(ts), UAS-egr | 48h 30ᵒC, D7 AED |
| Fig. S6 | R, V, W | ; 10xStat92E-dGFP | 18ᵒC, till D7 AED |
| Fig. S6 | S, V | enGAL4, UAS-mRFP.NLS/UAS-Ptp61F RNAi(i2-5); 10xStat92E-dGFP | 18ᵒC, till D7 AED |
| Fig. S6 | T, W | enGAL4, UAS-mRFP.NLS; UAS-Socs36E-RNAi(HMS01450)/10xStat92E-dGFP | 18ᵒC, till D7 AED |
| Fig. S6 | X | UAS-GFP; rn-GAL4, tub-GAL80(ts) | 12h 30ᵒC, D7 AED |
| Fig. S6 | Y | UAS-GFP; UAS-Stat92E-3xHA/rn-GAL4, tub-GAL80(ts) | 12h 30ᵒC, D7 AED |
| Fig. 7 | A, G | TRE-RFP; rn-GAL4, tub-GAL80(ts), UAS-egr | 24h 30ᵒC, D7 AED |
| Fig. 7 | B, G | TRE-RFP; UAS-Stat92E-3xHA/rn-GAL4, tub-GAL80(ts), UAS-egr | 24h 30ᵒC, D7 AED |
| Fig. 7 | C, H | UAS-GFP; rn-GAL4, tub-GAL80(ts), UAS-egr | 24h 30ᵒC, D7 AED |
| Fig. 7 | D, H | UAS-GFP/UAS-Ptp61F RNAi (i2-5); rn-GAL4, tub-GAL80(ts), UAS-egr | 24h 30ᵒC, D7 AED |
| Fig. 7 | E, I | 10xStat92E-dGFP; rn-GAL4, tub-GAL80(ts), UAS-egr | 24h 30ᵒC, D7 AED |
| Fig. 7 | F, I | 10xStat92E-dGFP; UAS-Socs36E-RNAi/rn-GAL4, tub-GAL80(ts), UAS-egr | 24h 30ᵒC, D7 AED |
| Fig. 7 | J, L, M | Ubi-GFP.E2f[1-230], Ubi-mRFP1.NLS.CycB[1-266]; rn-GAL4, tub-GAL80(ts) | 24h 30ᵒC, D7 AED |
| Fig. 7 | K, L, M | Ubi-GFP.E2f[1-230], Ubi-mRFP1.NLS.CycB[1-266]; UAS-Stat92E-3xHA/rn-GAL4, tub-GAL80(ts), UAS-egr | 24h 30ᵒC, D7 AED |
| Fig. 7 | N | UAS-GFP; rn-GAL4, tub-GAL80(ts), UAS-egr | 24h 30ᵒC, D7 AED |
| Fig. 7 | O | UAS-Stat92E-3xHA/rn-GAL4, tub-GAL80(ts), UAS-egr | 24h 30ᵒC, D7 AED |
| Fig. 7 | P | UAS-GFP; rn-GAL4, tub-GAL80(ts), UAS-egr | 24h 30ᵒC, D7 AED |
| Fig. 7 | Q | UAS-Ptp61F RNAi (i2-5)/rn-GAL4, tub-GAL80(ts), UAS-egr | 24h 30ᵒC, D7 AED |
| Fig. 7 | R, S | TRE-RFP; UAS-FLP.Exel, Ubi-p63E(FRT.STOP)Stinger’/rn-GAL4, tub-GAL80(ts), UAS-egr | 24h 30ᵒC, D7 AED, R0h or R48h |
| Fig. S7 | A | UAS-GFP; rn-GAL4, tub-GAL80(ts) | 24h 30ᵒC, D7 AED |
| Fig. S7 | B | UAS-GFP; UAS-Stat92E-3xHA/rn-GAL4, tub-GAL80(ts) | 24h 30ᵒC, D7 AED |
| Fig. S7 | C | UAS-GFP; rn-GAL4, tub-GAL80(ts), UAS-egr | 24h 30ᵒC, D7 AED |
| Fig. S7 | D | UAS-GFP; UAS-Stat92E-3xHA/rn-GAL4, tub-GAL80(ts), UAS-egr | 24h 30ᵒC, D7 AED |
| Fig. S7 | E | Ubi-GFP.E2f[1-230], Ubi-mRFP1.NLS.CycB[1-266]; rn-GAL4, tub-GAL80(ts) | 24h 30ᵒC, D7 AED |
| Fig. S7 | F | Ubi-GFP.E2f[1-230], Ubi-mRFP1.NLS.CycB[1-266]; rn-GAL4, tub-GAL80(ts), UAS-Stat92E | 24h 30ᵒC, D7 AED |
| Fig. S7 | G | UAS-GFP; rn-GAL4, tub-GAL80(ts) | 24h 30ᵒC, D7 AED |
| Fig. S7 | H | UAS-Stat92E-3xHA/rn-GAL4, tub-GAL80(ts) | 24h 30ᵒC, D7 AED |
| Fig. S7 | I | UAS-Ptp61F RNAi (i2-5); rn-GAL4, tub-GAL80(ts) | 24h 30ᵒC, D7 AED |
| Fig. S7 | I | rn-GAL4, tub-GAL80(ts), UAS-egr | 24h 30ᵒC, D7 AED |
| Fig. S7 | I | UAS-Ptp61F RNAi (i2-5); rn-GAL4, tub-GAL80(ts), UAS-egr | 24h 30ᵒC, D7 AED |
| Fig. 8 | A | TRE-RFP; 10xStat92E-dGFP/rn-GAL4, tub-GAL80(ts) | 44h 30ᵒC, D6 AED |
| Fig. 8 | B | UAS-ras[v12]/TRE-RFP; 10xStat92E-dGFP/rn-GAL4, tub-GAL80(ts) | 44h 30ᵒC, D6 AED |
| Fig. 8 | C, I | UAS-scrib RNAi/TRE-RFP; 10xStat92E-dGFP/rn-GAL4, tub-GAL80(ts) | 44h 30ᵒC, D6 AED |
| Fig. 8 | D-F, J, K | UAS-ras[v12],scrib RNAi/TRE-RFP; 10xStat92E-dGFP/rn-GAL4, tub-GAL80(ts) | 44h 30ᵒC, D6 AED |
| Fig. 8 | G | UAS-scrib RNAi/Ubi-mRFP1.NLS.CycB[1–266]; 10xStat92E-dGFP/rn-GAL4, tub-GAL80(ts) | 44h 30ᵒC, D6 AED |
| Fig. 8 | H | UAS-ras[v12],scrib RNAi/Ubi-mRFP1.NLS.CycB[1–266]; 10xStat92E-dGFP/rn-GAL4, tub-GAL80(ts) | 44h 30ᵒC, D6 AED |
| Fig. S8 | A | 10xStat92E-dGFP/rn-GAL4, tub-GAL80(ts) | 48h 30ᵒC, D6 AED |
| Fig. S8 | B | UAS-scrib-RNAi; 10xStat92E-dGFP/rn-GAL4, tub-GAL80ts | 48h 30ᵒC, D6 AED |
| Fig. S8 | C | TRE-RFP; 10xStat92E-dGFP/rn-GAL4, tub-GAL80(ts) | 44h 30ᵒC, D6 AED |
| Fig. S8 | D | UAS-scrib RNAi/TRE-RFP; 10xStat92E-dGFP/rn-GAL4, tub-GAL80(ts) | 44h 30ᵒC, D6 AED |
| Fig. S8 | E, G | UAS-ras[v12],scrib RNAi/TRE-RFP; 10xStat92E-dGFP/rn-GAL4, tub-GAL80(ts) | 44h 30ᵒC, D6 AED |
| Fig. S8 | F | TRE-RFP; 10xStat92E-dGFP/rn-GAL4, tub-GAL80(ts), UAS-egr | 44h 30ᵒC, D6 AED |
| Fig. S8 | H | Ubi-mRFP1.NLS.CycB[1–266]; 10xStat92E-dGFP/rn-GAL4, tub-GAL80(ts) | 44h 30ᵒC, D6 AED |
