# Supplementary material for: Mutual repression between JNK/AP-1 and JAK/STAT stratifies senescent and proliferative cell behaviors during tissue regeneration
Source: PLoS Biol. 2023 May 30;21(5):e3001665. doi: 10.1371/journal.pbio.3001665 (PMC10228795; doi:10.1371/journal.pbio.3001665)

File S5 Original Western blot images for data shown in Fig S6.1 D.

Western Blot 020218 - 680 nm channel – anti-GFP (STAT92E-GFP and HP1-GFP)

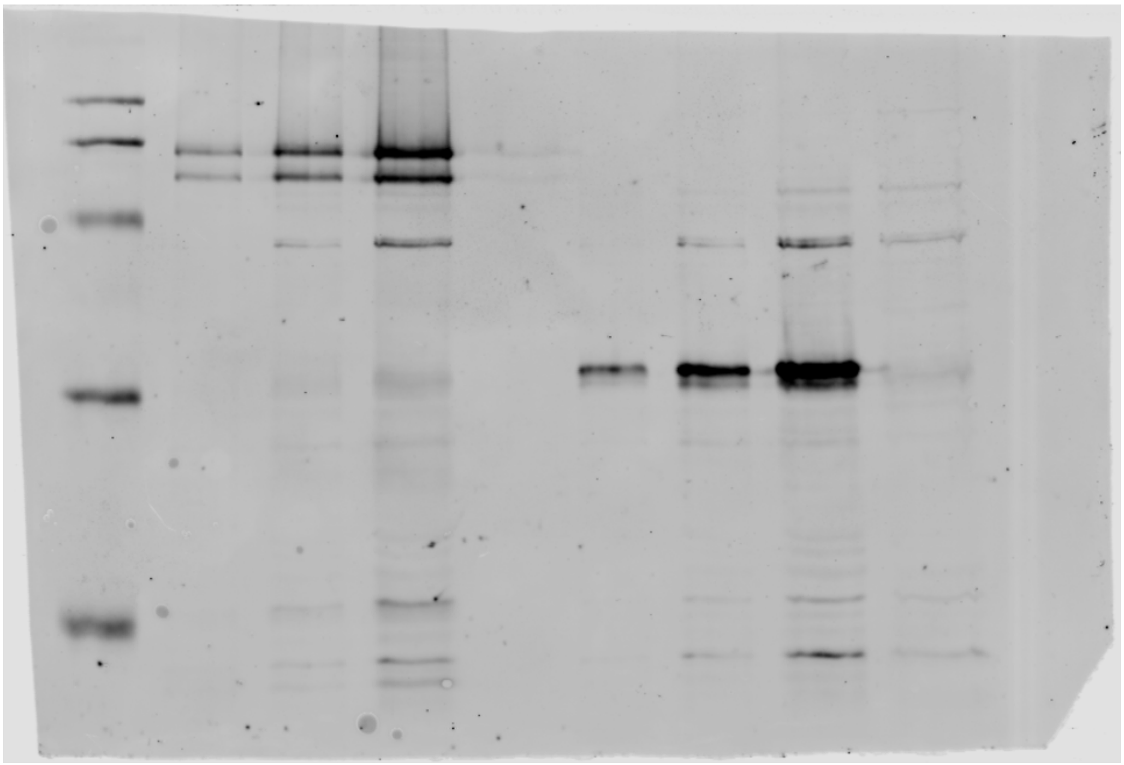

Western Blot 020218 - 800 nm channel – anti-tubulin

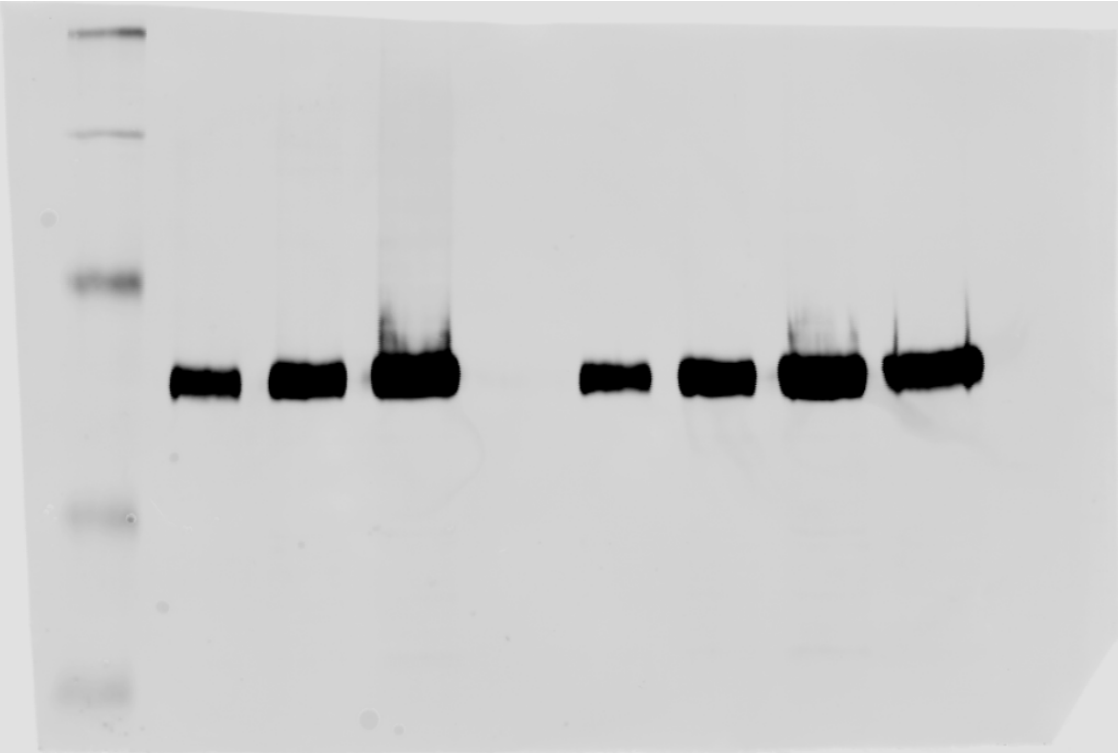

Supplement: S1 Raw Images — Original western blot images for data shown in S6D Fig. (PDF) [file pbio.3001665.s015.pdf]
