# Supplementary material for: Mutual repression between JNK/AP-1 and JAK/STAT stratifies senescent and proliferative cell behaviors during tissue regeneration
Source: PLoS Biol. 2023 May 30;21(5):e3001665. doi: 10.1371/journal.pbio.3001665 (PMC10228795; doi:10.1371/journal.pbio.3001665)
Supplement: Attachment — Submitted filename: 001 Response to reviewer.pdf [file pbio.3001665.s016.pdf]

To all reviewers

We would really like to thank you for your careful consideration of our work and are grateful for your thoughtful comments. In response to your concerns, we have drastically reworked the basic structure and modified the focus of the manuscript. We restructured the introduction and the entire flow of the manuscript to facilitate better access to our story. We added new experimental data, quantifications and modeling to strengthen the core concept of JNK-mediated cell-autonomous repression of JAK/STAT signaling and the consequences for tissue repair. We eliminated peripheral data and reorganized the existing data, together with new experimental and modeling data, into new Figures 1-9. The extensive changes to the text were documented in two successive 'track-changes' files submitted with this revision. We also submitted a data repository file to document our data quantification results. In addition, we include extensive documentation of our replicate and sample numbers in our figure legends (also for data not quantified in detail).

New experimental and modeling data sets are now located in these figures:

- Fig 1 A-E,I-S, U,W,V,X
- Fig 2 C, F, I, L, O, R
- Fig 3 I-M
- Fig 4 F-L
- Fig 5 B', B'', C', C'', D', D''
- Fig 6 D-F, H
- Fig 7 E,F, I, R,S
- Fig 8 G,H

- Fig S1 D,E
- Fig S2 C-K
- Fig S3-2 A,B
- Fig S4 D-G
- Fig S5.1 all panels
- Fig S6.3 A-F, I
- Fig S7 I
- Fig S8 H

Please also consider our detailed responses to individual concerns below:

REVIEWER 1:

Main comments

The manuscript by Classen and colleagues describes a molecular mechanism underlying the segregation of cell behaviors during regeneration. They start with their and others' observations that cells engaged in JNK/AP1 signaling, identified with TRE-RFP and generally stalled in G2, segregate to different areas of the Drosophila wing disc than cells engaged in JAK/STAT signaling identified with *STAT92E*-GFP. Because JNK signaling cells transcribe the Upd ligands that activate JAK/STAT, they ask why aren't JNK cells also positive for *STAT92E*? They find that cells begin expressing both reporters, but over 24h of JNK activation, they lose JAK/STAT signaling. They next develop a mathematical model to reproduce the JNK-induced activation of JAK/STAT non-autonomously while simultaneously inhibiting JAK/STAT autonomously. The model suggests that mutual repression of JNK by JAK/STAT and of JAK/STAT by JNK is sufficient to explain the results, so they next look for the

mechanisms underlying the repression. Using co-expression and gene knockdown, they report that the JAK-STAT induced transcription factor Zfh2 inhibits the JAK/STAT pathway; and the phosphatase *Ptp61F*, which is upregulated by JNK, inhibits the JAK/STAT downstream transcription factor, *Stat92E*. Returning to cell behaviors, they report that when cells are forced to activate both pathways, apoptosis increases as the G2 arrest is lost and these cells now enter the cell cycle. They conclude by examining a genetically induced tumor of the wing disc and showing that JAK/STAT and JNK signaling segregate in this tissue also, as they do in the non-tumorous wing discs.The conclusion of the study is that JNK signaling associated with wounds is sufficient to induce two segregated populations of cells with different signaling pathways and different cell behaviors. This conclusion is interesting, novel, and identifies a mechanism to address the important question about how wounds pattern the cell responses around them.

However, the paper is very hard to read, and the data are hard to evaluate because they are poorly presented. Further, there is a lot of over-interpretation; re-writing sections with a lighter tough, acknowledging incomplete answers, is required. About the paper being hard to read: First, it is in a strange style, with the figures rigidly forming the sections of the results, as though it were a poster. The text and the figure legends are each, and sometimes both together, insufficient to understand the figures. The order of the figures is also hard to make sense of, compounded by the frequent references to supplementary figures.

- We appreciate this criticism and hope that our extensive revision addresses these concerns. We rewrote all sections with a lighter touch, we toned down the language and interpretation of our results. We may have been overthinking the structure of the manuscript to get our point across – we hope that the revisions may provide a nicer story-telling flow.
- We agree that there is a lot of supplemental data that impedes readability. They are there because: (1) We needed to exclude alternative models and interpretations. (2) We needed to provide experimental controls (i.e. test constructs for functionality). We cannot write ‘data not shown’ to describe these experiments and controls. We have now tried to slim them down but requests from 3 other reviewers needed to be considered as well. (We would be absolutely happy to eliminate supplemental data if you feel they do not need to be shown because the controls are not necessary.)

About the data: it is difficult to evaluate how much reproducibility and variability have been assessed. A great number of figure panels in this study show a single example, and it's not clear how to interpret the assurance in the methods that n is at least two for every experiment when it's not addressed in the text, figure, or legend. For example, the only data addressing reproducibility and variability in Fig. 1 is Fig. I,J; even panels M,N measure only a single sample. Further, 1I and J are difficult to understand and represent mostly background levels - why not remove background before assessing the levels of the reporters? The same is true for all of Fig. 2, for Fig 5 I,J,O,P; and most of the extensive supplemental data (n=2 is not enough for statistics, as done in Suppl Fig. 1.2). Further, there is no indication that samples were scored blinded. This is a particular concern when there is so much uniqueness in how image data is manipulated - sometimes a section, sometimes a projection, sometimes thresholded, despeckled, denoised, etc. Such individualized image analysis for each experiment can open the door to unconscious bias, which is why blinded analysis is so important.

Please allow us to address these points together:

- We now provide additional and more intuitive quantifications for our data sets to address reproducibility and variability. For example, in Figures 2, S2.1, 3, S3.2, 4, 6, S6.3 or 7. In addition, we provide statements for replicates and sample size in figure legends specifically for

data sets that have not been quantified in detail. We hope that these changes provide a sense for the reproducibility and robustness of our data.

- We reduced S1.2 (now S3.1) to n=1. We still include the line trace to illustrate the data.
- We removed Figure 1I and J from the paper and now provide more direct quantification of JAK/STAT reporter activity.
- We only applied thresholding, despeckling and denoising to generate area masks for segmentation in FIJI (for example to generate a mask of the entire disc area by using the DAPI channel to determine wing disc size). We did not manipulate images with these functions to measure fluorescence reporter activity. None of the IF images shown in the manuscript have been thresholded, despeckled or denoised. Only the same brightness and contrast adjustments were made to control and experimental discs for better visualization.
- We are showing single sections for most images now.
- We generally agree with your comment on scoring samples blindly. This is an important point that we have carefully considered during all our image analysis workflows. Scoring blindly is particularly crucial when humans provide the score. Yet, we have consistently created automated analysis workflows to avoid potential bias and the exact same workflow was applied to all samples (including all functions for generating segmentation masks).

Some examples of over-interpretation:
*Lines 148-150, "We show that upd cytokines activate JAK/STAT only non-autonomously in a cell population spatially segregated from high JNK-signaling and upd-producing cells." The authors do not show that upd cytokines are even synthesized and secreted, let alone that they activate JAK/STAT. Although it is a fair inference, it is not "shown".
*In lines 186-188, "Altogether, these experiments demonstrate that tissue damage associated with high levels of JNK/AP-1 signaling repress JAK/STAT activity in a cell autonomous manner, independent of developmental competence." It is clear that the overexpressed levels of JNK/AP1 are repressing JAK/STAT, but not that it's the "tissue damage".
* In Fig. S3, the difference in MMP1 levels +/- Zfh2 may be statistically significant, but it is certainly not a large effect. The Y-axis is misleading as it does not start at 0 and so exaggerates the effect. This small effect cannot support the strong statements in the abstract or discussion about how JAK/STAT suppress JNK activation via Zfh2 - tone it down.
* The main takeaway of the manuscript is that JNK signaling can self-organize the patterning of cell behaviors, G2-stall or proliferation, each mediated by its own pathway. However, the only place that proliferation is quantified with respect to Jak/Stat signaling is in tumors, Fig.6I. Although the effect may be significant, it is not large (perhaps a mean of 2.3 to a mean of 2.9).
*In line 391, there is no evidence here that segregation of signaling networks provides an advantage to tumors.

- We agree that our language was not always well chosen for these interpretations. We addressed your concerns in the following manner:
- We rewrote the manuscript and made sure that our language stayed close to our experiments.
- We restructured the introduction and included stronger referencing of existing literature to provide context for our conclusions.
- We provide additional quantifications of experimental data or clear references to papers where relevant processes have been previously quantified (for example, we recently described and quantified regenerative proliferation in [1]).

In line 298/Fig 4J, co-expressing *Stat92E* with *egr* does not demonstrate that dephosphorylation of *Stat92E* is rate limiting, only that *Stat92E* is rate limiting. Can you restore normal reporter levels by co-expressing *Ptp61F* along with *egr* and stat93E-HA?

- We removed this peripheral conclusion from the manuscript. We now also provide new experimental data, that SOC36E represses JAK/STAT activation in JNK-signaling cells (new Fig 6 and S6.3), making other modes of JAK/STAT suppression possible.
- However, to specifically address this comment, we also performed the following experiment: We tested a UAS-line generated by Martin Zeidler, which expresses the predicted nuclear isoform of *Ptp61F* (w; p[w+, UAS-*Ptp61F*c]5a.3/TM3). We find that expression of this isoform using *en*-GAL4 appears to be lethal, as we cannot recover any larvae. Given the additional risk this finding imposes on the proposed rescue experiment, we did not pursue a recombination genotype for all our constructs on the 3rd chromosome (the *egr*-expression system, the UAS-*Ptp61F* and UAS-*STAT-HA* are all on chromosome 3). The time frame for generating and testing the recombinant chromosome, and performing a well-controlled experiment was outside the possibilities of this revision.

Is it possible the G2 stall cells are endocycling? Their nuclei look significantly bigger than surrounding nuclei.

- Indeed, these nuclei are bigger. However, their DNA density (DAPI intensity) is reduced at the same time. We are currently investigating the mechanisms underlying nuclear expansion and chromatin regulation in a separate project in the lab. We observe regulation of nuclear envelope proteins and histone modifications in JNK/AP-1 signaling cells.
- We also kindly refer to these two papers, where we show that proliferating and arrested cells in the regenerating disc are not endocycling [1, 2]. Please note that arrested cells fail to incorporate EdU, suggesting that DNA replication does not take place (see also new Fig 1).
- We would also like to point out, that regenerative endoreplication or polyploidization has been described for post-mitotic or already endoreplicating cells (adult gut enterocytes, follicle cells, embryonic ectoderm) [3-6]. In contrast, the imaginal disc is a highly mitotic, exclusively diploid tissue at the time of tissue damage. There is a strong requirement for imaginal discs to maintain a diploid state during regeneration to ensure that these discs can successfully differentiate and change shape into adult structures (for example photoreceptors in eyes, wing hairs in wings, or sensory bristles on legs). Polyploidy would disrupt differentiation processes into adult cell types, and the larger size of polyploid cells would disturb morphogenesis of eye, leg and wing tissue during pupal metamorphosis. Moreover, multiple labs which have studied imaginal disc regeneration have not reported polyploidization. We thus think that it is unlikely that endoreplication or polyploidization plays a significant role in imaginal disc regeneration, in general.

Minor comments

1. Please reserve the ">" (greater than) symbol for Gal4-driven gene expression, and do not use it for "TRE>RFP" and "*STAT92E*>GFP". Instead use "TRE-RFP" and "*STAT92E*-GFP". Otherwise, it appears you are using rnGal4 to drive Eiger and TREGal4 to drive RFP and *STAT92E*gal4 to drive GFP, all at the same time.

- This has been corrected in all figures, figure legends and manuscript.

2. The 2° antibodies used for the immunofluorescence experiments are not described.

- All secondary antibodies were added Table S1 Key Resources Table.

3. Show rn>GFP in Fig. 1, for readers not familiar with this domain.

- Done.

4. Could the authors double-check that the DAPI image in 1A is not (accidentally) the same disc shown in 1C?

- We checked and it is not the same disc.

5. The authors should cite the Park et al NCB 1997 (Greco) paper showing spatial segregation of cell behaviors around wounds.

- Absolutely, we mentioned it to the editor but did not include it in the first draft. It is now cited in the discussion.

6. What are the diagonal yellow arrows in Fig. 1?

- This represents the axis along which the different reporter profiles were measured from the pouch centre (PC) to the disc periphery (DP). We now clarify this in the figure legend for the new Fig 3.

7. Line 167, referring to Fig. S2A seems to be an error.

- Fig. S2A displayed the *control* disc for Fig. 2C (RFP-expressing clones not expressing hep^{act}, located in the peripodium).

REVIEWER 2:

Jaiswal and colleagues report mutually exclusive activities of JNK and JAK/STAT during regeneration and tumor growth in Drosophila wing discs. Both of these pathways are activated in the context of tissue damage in a number of different experimental systems, and are required for regeneration. This study addresses their spatial and temporal organization. Overexpression of Eiger (Drosophila TNF) activates JNK cell autonomously and ,JAK/STAT non-autonomously. This group has shown in a 2019 eLIFE paper that cells that activate JNK arrest in G2 and this arrest provides a pro-survival benefit. The current manuscript reports that activation of JNK and STAT are mutually exclusive such that STAT is not activated in cells with high JNK activity. Depletion of a phosphatase encoded by *Ptp61F* in cells with active JNK leads to STAT activity in these cells, leading the authors to conclude that *Ptp61F* is the reason for the lack of STAT activity. Mutual exclusion is observed also with ectopic activation of JNK with a constitutively active kinase, which is sufficient to inhibit STAT activity even in cells that normally shows high developmental STAT activity. A similar spatial relationship is seen in tumors driven by a combination of oncogenic RasV12 and the loss of polarity through scrib mutations. Forced co-activation of JNK and STAT resulted in cell death. The authors propose that self-organized, mutually exclusive expression of JNK and JAK/STAT allows JNK-active cells to survive and STAT-active cells to proliferate during regeneration and in tumor growth.

JNK and JAK/STAT are conserved signaling pathways with clear importance for development, tissue regeneration and cancer. Understanding how they corporate in tissue regeneration or in tumor growth is a significant goal. A self-organizing and mutually exclusive arrangement of JNK and JAK/STAT activities is of potential interest to readers of PLoS Biology. I have several reservations about the approach and data interpretation that should be addressed first, however. For example:

Main comments

General concern 1.

The authors use signal area in many of their quantifications (e.g., Fig. 5D). This is a usual practice for wing discs and works well because they made up of single cell layers. The problem here is that experimental manipulations used changed tissue organization to result in 3D folds (e.g., Fig. 5B). How do the authors justify using 2D areas to quantify signals in tissue that is clearly folded in 3-dimension?

- This is a completely valid point. From our previous work, we know that a 2D projection yields a good approximation of apoptotic activity in 3D. However, to directly address your concern, we have repeated all measurements as 3D volume quantifications and included these in the manuscript. Our conclusions remain unchanged, if compared to our 2D quantifications.

General concern 2.

Overexpression of eiger in the pouch is a standard technique used to ablate the pouch. That means the disc regions being analyzed are a mix of living and dead cells whereas control discs will have only living cells. So changes observed could be an indirect consequence of cells dying. To give a specific example, Fig. 1 shows convincingly that JNK and dynamic STAT reporter activities are mutually exclusive and that EdU incorporation and STAT reporter activity are reduced in JNK-active cells. Could that be a simple consequence of JNK-active cells dying? Similarly, an alternate explanation for Fig 2G-J is that as JNK activity in the pouch increases, cells start to die and no longer incorporate EdU. Knowing what fraction of the pouch is made of live/dead cells at R0 and focusing the analysis only to live cells could address these concerns.

- We agree that this could be a concern and have considered this as well. We thus have addressed this issue with our methods and experiments:
- We focused our analysis on viable cells. To this end, our experiments included (in almost all cases) a DAPI staining as a proxy for cell viability. In our analysis, we selected medial sections and sample areas where pycnotic nuclei were as rare as possible, which is also why we show DAPI stainings in many of our figures.
- In addition, we more directly tested if cell death pathways repress JAK/STAT activity in *egr* or hep-expressing cells. Yet, neither a reduction in RHG-proteins (heterozygosity for the *rpr,hid,grim* deficiency Df(3L)H99) and thus reduced activity of initiator Caspases (i.e. Dronc) nor inhibition of more downstream regulators (i.e. Drice or Dcp-1) by expression of p35, reversed the repression of JAK/STAT activation in JNK-signaling cells (see new Fig. 4 and S4).
- In addition, our data on viable tumors in Fig. 8 argues that our observations are not just simply arising from cell death or activation of cell death machineries.
- So, while we cannot exclude that JAK/STAT signaling may be regulated in certain instances by the cell death machinery, we have not found any evidence that activation of apoptotic cell death pathways per se is initially required for JNK-mediated repression of JAK/STAT.

Specific concerns and recommendations

1. Fig. 2A-C provides the most convincing evidence that cells with ectopic JNK activity repress STAT within themselves but activate STAT in the neighbors. These cells are also 'undead' due to co-expression of p35. Is it possible to make undead cells without using JNK and ask if you see a similar effect on STAT? That would be a nice control.

    - We agree and have performed an analysis of wing disc expressing either *scrib-RNAi* or *scrib-RNAi,p35* under the control of *rn-GAL4*. Both experiments are consistent with our conclusion and are now included in Fig. 4L and Fig. S4 F,G.

2. Fig. 2F. I cannot tell how the A/P boundary determined here. If JNK is being activated in the P compartment and JNK-high cells are supposed to inhibit STAT, why is STAT-GFP showing up here? I see a lot of pyknotic (small condensed) nuclei in the supposed A compartment even though JNK is activated only in the P compartment. To demonstrate across-compartment activation, it would help to see the whole disc with the compartments clearly defined.

- We find that the *en-GAL4* driver is most active in the pouch, as can be seen by expressing a UAS-RFP construct (see image below), thus JNK activity is highest there. We conclude that the activity in the hinge is not high enough to really suppress JAK/STAT. Moreover, according to our model JAK/STAT activity in the hinge could dampen JNK activation there. This is why we included MMP-1 staining to really show where JNK is active in this genotype because this is the ultimate determinant of bistable pattern formation.
- We have included an UAS-RFP construct in the cross to show the compartment outline and show a larger view of the disc (new Fig. 3H).

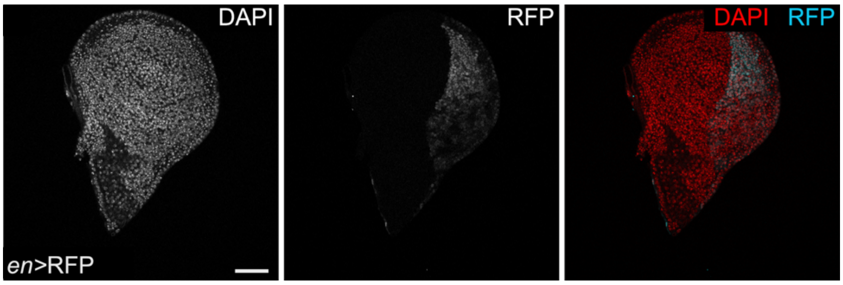

3. Fig. 2K-M. First, the DAPI panels are showing STAT>dGFP. In these, GFP in the pouch seems to first increase from K to L then decrease from L to M. Again, this could be an indirect consequence of pouch cells dying.

- We corrected the labeling.
- We tested the role of apoptotic cell death (now Fig. 4 and S4-1) and performed our analysis using the criteria described in response to General concern 2.

4. Fig. 4. The increase in STAT>GFP reporter from B to C is not impressive. It is also unclear how the authors know to compare the area just within the yellow circle. They should first identify the JNK-active area in an unbiased manner and then make the comparison. If they want to make the conclusion that the mechanism by which JNK represses STAT is through *Ptp61F* (as shown in Fig. 7C), they need to connect JNK to *Ptp61F* in their experimental system rather than cite the work of others that address this in other contexts (ref 23, 116). Without such data, the authors cannot rule out the possibility that *Ptp61F* simply sets a threshold for STAT activity and that JNK acts through another target to overcome it. And an alternate explanation for Fig. 4I that shows increased STAT>dGFP is that it is due to elevated overall STAT from STAT-HA expression, not that extra STAT is saturating *Ptp61F* (line 299-300). Can the authors rule this out?

- We apologize for not explaining it better. The yellow circle was just included to broadly guide the reader to the domain of interest, and was not the region we used to make the quantification. We use a consistent method with measuring the very central cells for JAK/STAT reporter activity, as is now clearly depicted in Figure 4J. The same method was used for these quantifications, and we are now highlighting these regions with a square to approximately depict the measured regions.
- As JNK can also be activated outside the *rn-GAL4* expressing cells, which do not experience the knock-down and are not the cells we need to quantify, identifying the JNK-signaling domain is not the right way quantify these experiments. We thus restricted our analysis to very central

cells, which we know are *rn-GAL4* positive from separate experiments using UAS-GFP. It is genetically and fluorescence-wise very tricky to combine all reporters, lineage tracers and UAS-constructs for these experiments. We thus chose to perform these experiments in this (best possible) way.

- We agree that the rescue of JAK/STAT activation by knock-down of one regulator is not 'striking', yet it is reproducible. We now provide evidence that also SocS36E is repressing JAK/STAT in JNK-signaling cells (new Fig. 6 and S6-3), thus multiple negative JAK/STAT regulators may contribute to this effect. While *Ptp61F* is mildly elevated, Socs36E is more strongly induced in *egr*-expressing disc (Fig. S6-3). This is also consistent with a direct JNK-mediated upregulation of Socs36E in other contexts of regeneration [7].
- We removed the suggestion that *Ptp61F* is acting directly on *Stat92E* because we cannot directly test it within the time frame of this project. As Socs36E represses JAK/STAT activation in JNK-signaling cells, other modes of JAK/STAT suppression are indeed possible.

5. Fig. 5. The nuclei in the boxed region in F are larger than those in the box in E and I wonder if the authors are looking at the peripordial cells instead of the columnar pouch. Again, 3D folding is apparent in these discs and could be confounding the analysis. The cell cycle data could also be interpreted in more than one way. For example, adding *Stat92E* to eiger increased the fraction of G1 cells, decreased the fraction of G2 cells, and increased EdU+ nuclei. The authors interpret this as *Stat92E* pushing the cells out of a (protective) G2 arrest by JNK. But the data are equally consistent with *Stat92E* stalling cells in G1 and prolonging S, without changing G2 (because what is being measured are % and rations that are relative and not absolute). Cell doubling times or mitotic index will be needed to distinguish between these possibilities.

- These are not peripodial cells. The high JNK-signaling, senescent cells have indeed larger nuclei. However, their DNA density (DAPI intensity) is reduced at the same time. We are currently investigating the mechanisms underlying nuclear expansion and chromatin regulation in a separate project in the lab. We observe regulation of nuclear envelope proteins and histone modifications.
- We agree with your possible interpretation of the cell cycle effects. Indeed, the cell cycle could still be stalled by extending other phases. However, we think that this is unlikely as we recently showed that JAK/STAT signaling is sufficient to speed up S-phase, and through this, could contribute to fast cell cycles during regeneration [1]. Our point here is, however, that the cells pass from a G2-arrest into G2/M and enter G1. This is the crucial conclusion: Because passing through G2/M provides the essential switch between being protected from apoptosis in the G2-arrest and gaining apoptosis competence in G1 by activation of p53 by the G2/M kinase Cdk1. This model is based on these two publications [2, 8] and we incorporate it repeatedly in the text (see for example Fig. S1A'). We now try to make that point more precisely in the writing of this section and our conclusions.

6. The authors conclude throughout the manuscript that cells with high JNK activity experience a 'a senescent-like G2 cell cycle stall' (line 118); 'their cell cycle is senescently arrested (line 37); acquire senescent phenotypes' (line 452). Yet, there are no data shown to demonstrate senescence such as staining for senescence markers.

- We now expanded the description of the senescent phenotype in the introduction and in Fig. 1 and 2, and provide data showing upregulation of the widely used senescence marker SA-beta-GAL in *egr*-expressing cells.

7. There are no data shown to support the conclusion or even address the possibility that '…cells with activated JNK/STAT suppress JNK activation via Zfh2' (line 30-31, Fig. 3B, Fig 7C legend).

- We originally showed this data in Fig. S3 A-C but have now removed it from the paper. Although analysed previously [9], the molecular mechanism of JNK repression by JAK/STAT is beyond the scope of this paper and not central to our main conclusion that JNK represses JAK/STAT.

8. Loss of cells to death seems to be missing in the modeling in Fig. 3. Cell death could make a difference if sustained JNK activity is needed to keep activating JAK/STAT. Loss of JNK active cells to death could cause a decline in JAK/STAT activity with time, making the unidirectional repression appear like bidirectional repression in Fig. 3D.

- I am not sure I completely understand this question and the logic of this concern. Could you rephrase this?
- If you are asking us to examine if low JNK activity: the probability of finding bistable solutions in the mutual repression model decreases with decreasing JNK activation rates (see Fig S5.2). This is consistent with our idea that a reduction in cell death correlates with successful tissue repair and the resolution of the bistable field.

REVIEWER 3:

Main comments
In my view, this work poses interesting questions in a powerful model system. The authors make a wise use of fly genetics to dissect the feedbacks between JNK/AP-1 and JAK/STAT. Here, modeling is fundamental to sort out the behaviour of such a bistable signalling network. Despite these positive premises, I find that the model system is not introduced clearly enough, which could be problematic considering the broad readership of PLOS Biology. The manuscript should also be improved in the presentation and use of the mathematical model. Most importantly, I feel that the conclusions of this work could be made stronger if the authors had used their time-courses of JNK/AP-1 and JAK/STAT activation to investigate the compartmentalization model.

Fig. 1

1) The authors over-express TNF-α/Eiger to induce wound healing and regeneration. I am not too familiar with this system and I have a hard time understanding how it works. I thought that over-expression of TNF-α/Eiger induces cell ablation in the center of the disc, through activation of JNK/AP-1. However, I gather from the introduction that JNK/AP-1 protect cells from cell death, although it activates inflammation pathways. Thus, I do not understand if Eiger over-expression induced cell death, protects cells from it or both at different times. I invite the authors to describe their model system more extensively, in particular clarifying what is the injury, what cellular processes occur during wound healing and which ones occurs during regeneration. Along similar lines, I invite the authors to provide legends within the Figures to guide the reader through the various reporters and perturbations that are used in this manuscript.

- We can see how this is confusing and apologize for not being more considerate of this. We have rewritten the introduction to describe how the two paradoxical behaviors caused by egr/JNK expression, namely cell death and protection from cell death, are linked to cell cycle progression. In short, the premise is the following (and is based on the existing literature cited in our manuscript) (see also Fig. S1A'): JNK arrests the cell cycle in G2 which protects cells from apoptosis, but competing inputs can activate cycling of JNK-signaling cells. This induces

apoptosis competence in G1 via activation of p53 and hid by the G2/M kinase Cdk1. The stressful environment of tissue damage may then cause p53/hid-mediated apoptosis of cells entering G1. Thus JNK can prevent apoptosis by G2-arrest but may also cause apoptosis by promoting hid transcription or cellular damage via ROS production, which can be read out by activated signaling competent p53 only in G1. In addition, we have restructured Figure 1 into new Figures 1 and 2 to give more space to the description of this cell population by markers for apoptosis, cell cycle and the senescent program. We hope that these changes provide a better basis for understanding the paper.

Fig. 2

2) The authors obtain time-courses of the patterning of JNK/AP-1 and JAK/STAT activation. I think that the presented results are interesting, as they show regions of transient co-activation of the two pathways. However, I am surprised that the authors did not analyse the implications of these temporal dynamics when building their regulatory network (see below). Can these dynamics be used to discriminate between different models and select parameters?

- We agree that this would have been nice. However, the precise parameters of many components in our model are essentially unknown and determining them would have to be an entirely new project beyond the scope of this manuscript. In addition, while we have a fairly good understanding of the behavior of our reporters, we did not vigorously determine their (linear) activity ranges, so it would be naive to correlate fluorescence levels with precise mathematical activities (at this point in time). Because of these considerations, we did not attempt to model our time series. Yet, we now provide time-resolved example of possible modeling solutions in the new version of the paper.

Fig. 3

The authors present in Fig. 3 a model of JNK/AP-1 and JAK/STAT compartmentalization. They compare two models: a "uni-directional repression model" and a "mutual repression model". The "mutual repression model" is a simple bistable switch and there are no surprises regarding its behaviour. Instead, the "uni-directional repression model" undergoes patterning because eiger expression is pre-patterned (bEIG(x)). Thus, the qualitative implications of the model are quite straightforward.

The authors use an "unbiased approach" to ask in which regions of the parameter space bistable patterns are generated. This approach is not described in detail. In addition, simulation results are not shown, so that we do not know how patterning occurs, how patterns differ for different parameter choices and what is considered a bistable pattern or not. The authors present a score table to show how often "bistable patterns" occur when varying different parameters (Fig. S3D), but I am not sure what to learn from the plot.

- We agree with this criticism and have extensively revised this section of the manuscript. We hope that our changes substantially alleviate some of the concerns. Specifically, we clarified our modelling strategy, provide simulation results and indicate the relationship between parameter values and bistability. The plot in S3D (now in S5-2) provides an analysis of the number of positive solutions obtained by individual parameter choices. It is thus a good global overview of which parameter range gives positive solutions and which system component is most sensitive to changes. This is best extracted from the bar graphs in the diagonal of the image. We revised our result section and figure legends to better explain this point.

I suggest the authors to better present and improve their modeling efforts as follows:

3) The authors should show simulation results (dynamics and final pattern), for different parameter choices. They should show representative patterns that are classified as bistable and not.

- We now include representative examples for final patterns of 'observed' bistable and 'simple' bistable cases. In addition, we visualize spatio-temporal dynamics for all used criteria, and for bistable and non-bistable patterns using kymographs (new Fig. 5 and Fig. S5-1).

4) The authors should describe the procedure used to explore the parameter space and score results.

- We have rewritten our materials and methods section, figure legends and results to provide clearer explanations of the parameter sampling and definitions of simple bistability and observed bistability.

5) The authors should compare the time evolution of JNK/AP-1 and JAK/STAT activation predicted in silico with the one obtained experimentally.

- Please consider our response to your main concern about Fig. 2 above. We feel that we do not have enough experimentally defined components available to really match the time-resolved dynamics of experimentally observed JNK and JAK/STAT activation with the large number of in silico solutions. We feel that this is beyond the scope of this project and may not change the basic conclusion that JNK-mediated repression of JAK/STAT is required for bistability.

6) The authors describe two qualitatively different patterns: the "simple bistable pattern" and the "observed bistable pattern". I do not think that "the observed bistable pattern" is a better fit of the experimental curves shown in Fig. 3A. For example, the authors point at JAK/STAT activation decreasing at the right boundary, but they do not emphasize JNK exhibiting larger variations on the left. I invite the authors to use a fitting or smoothening procedure to obtain an experimentally observed reference pattern; then, they would compare their model results to that experimental curve, without the need of postulating an alternative "simple bistable system".

- To address your concern, we analysed the entire data set again. We updated the observed bistability criterion as a strong "positive correlation with the shape of the experimental curve" for both the JAK/STAT and JNK gradients. This returned solutions that were aimed to be close to the experimental observations. However, when applying this correlation criterion, we noticed that narrow JNK gradients are not classified as bistable. Therefore, we additionally kept the analysis using 'simple' (descriptive) criteria to extract bistable solutions.
- When we combined both selection methods (coefficient matching for observed bistable patterns and descriptive parameter selection for simple bistable patterns), our sampling produced the most intuitive results. We thus present all 3 approaches in new Fig. 5, as they also illustrate the short-comings of unbiased matching of the experimentally determined patterns.
- Again, we updated the relevant methods sections to explain the scoring methods.

7) The authors should investigate what is the importance of the gradient of eiger production bEIG(x). Is that needed for bistability? Is the feedback system simply amplifying the differences between the domains that have high and low bEIG(x)?

- This is indeed an interesting question. We looked it from several perspectives and find evidence that the gradient of eiger is not affecting the output of the system per se. For example, for parameter combinations classified as bistable, there is no strict relationship between the width, amplitude or integral of the Eiger gradient, and the width of the JNK gradient or the intersection point of JAK/STAT and JNK (see Fig S5.1 I-K). Thus, *egr* concentrations appear to not be

crucial. Yet, how *egr* is interpreted appears to be important. One of the central parameters that determines bistable patterns is JNK activation level as well as activation rates. We now include these results in Fig. S5-1 and this conclusion is also reflected in Fig. S5-2.

- As for the feed-back system: the feedback loops would certainly amplify the differences between the domains of high and low bEIG(x). However, because we do not determine experimental values for negative (not included) and positive feedback (for example dome receptor upregulation by JAK/STAT) parameter in the system, we refrain from making a statement about their necessity or the strength of the individual loops.

8) In the paragraph starting at line 257, the authors say "the length scale-independent space utilized by the model raises the possibility that this mutual repression network could act at length scales ranging from neighboring cells to multicellular tissues." I think that this sentence is misleading, as the system has various characteristic lengths that can be obtained by taking the square root of any diffusion constant divided by any degradation rate. Unless those parameters change accordingly, the model is not scale-independent.

- We understand your concern, although it is less clear how this may work out in non-linear systems. However, we removed this specific statement from the manuscript as we did not explore this aspect further. If necessary, we could add the following statement to the manuscript to discuss the spatial properties of the model like this:

  "Because our modeling approach considers space on a relative length scale (from 0-100), the model does not consider discretization by different cells, i.e., no parameter combinations were excluded where the regulatory network model acts on length scales of neighboring cells, or even on length scales of the entire multicellular tissue."

Figure 5

9) The authors show that the activation of JAK/STAT in cells having active JNK/AP-1 leads to excess apoptosis. Do the authors think that this phenotype is cell-autonomous or does it result from the perturbation of the spatial pattern of JNK/AP-1 and/or JAK/STAT? Can the authors achieve an experimental condition in which JAK/STAT is active at the center of the disc and JNK/AP-1 at its periphery? Does this altered pattern impair cellular behaviour and regeneration?

- This is an important idea and we have performed 2 experiments to address this, which are included in Figure 3 H and Fig. S3.2 A,B. In the first experiment (using *en-GAL4*) we can activate JAK/STAT in the central anterior pouch and JNK in the posterior pouch. In the second experiment, we use (the weaker) *A30-GAL4*, which is a wing disc hinge driver. JAK/STAT is developmentally active in the hinge. When we express *egr* in the hinge, we still get bistable patterns: where JNK is high, JAK/STAT is low; where JAK/STAT is high, JNK is low. We interpret this pattern to reflect mutual repression between both pathways in a domain where JAK/STAT already has high basal activity.

REVIEWER 4:

Main comments

In my opinion the main limitation of this paper in its current form is the lack of comprehensive description of the model system being used. The authors present the Eiger over-expression wing disc model as a 'extensively used' model to study tissue regeneration following damage. To understand the implications

of (and interpretation of) data in this manuscript, the Introduction would benefit from more background on the model being studied.

For example, does Eiger over-expression drive loss (i.e. death) of some wing disc cells or is the Eiger-induced 'damage' not lethal? In the introduction they write "We recently reported that high JNK/AP-1 signaling facilitates survival in wounds and tumors by mediating a cell cycle stall in G2 which is characterized by anti-apoptotic and senescent features [31]". Yet they see upregulation of Dcp1 in the JNK domain in Figure 1B (which seems a paradox). Given this Dcp1 staining it indeed appears after 24h that cell death is occurring throughout most of the JNK domain but this is not directly mentioned in the accompanying text. This would be useful information to understand how similar this model is to a more traditional 'wound'. If JNK+ cells do eventually die, it would also be informative to include a timeline of this death (over 7h, 14h and 24h) and an indication of how long these cells will continue secreting Upd?

- We apologize for not introducing our system well and for not giving enough room to the premise of the paper. We can see how the different effects of JNK are confusing.
- Therefore, we have rewritten the introduction to describe how the two paradoxical behaviors caused by egr/JNK expression, namely cell death and protection from cell death, are linked to cell cycle progression. In short, the premise is the following (and is based on the existing literature cited in our manuscript) (see also Fig. S1A’): JNK/AP-1 arrests the cell cycle in G2 which protects cells from apoptosis, but competing inputs can activate cycling of JNK-signaling cells. This induces apoptosis competence in G1 via activation of p53 and hid by the G2/M kinase Cdk1. The stressful environment of tissue damage may then cause p53/hid-mediated apoptosis of cells entering G1. Thus, JNK/AP-1 can prevent apoptosis by G2-arrest but may also cause apoptosis by promoting hid transcription or cellular damage via ROS production, which can be read out by activated signaling competent p53 only in G1.
- In addition, we have restructured Figure 1 into new Figures 1 and 2 to give more space to the description of the JNK-signaling cell population by markers for apoptosis, cell cycle and the senescent program. We included a time series of apoptosis. We hope that these changes provide a better basis for understanding the paper.
- We also include in this response, a time series of Upd3.1 LacZ activity during *egr*-expression (top figure) and into recovery 24 h after *egr*-expression (figure panels below). Upd3.1 LacZ activity closely scales with JNK activity throughout, which is not a major surprise. We now also added data to show that *rn-GAL4 egr*-expressing cell transcribe Upd1, 2 and 3 (Fig. S2-1). This does not directly answer your question about how long dying cells will secrete Upds. However, we think that we can closely correlate *upd* expression with *egr*-expression.

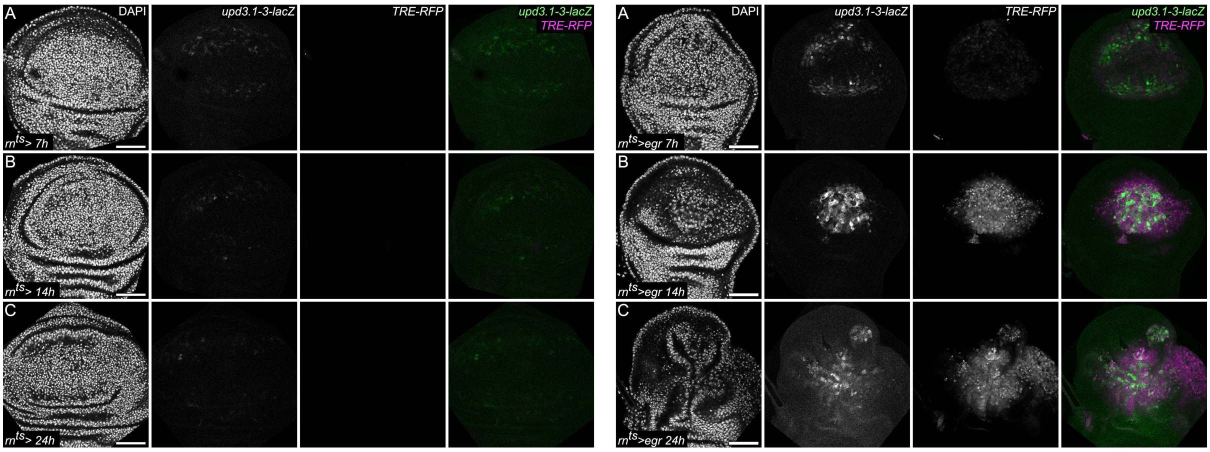

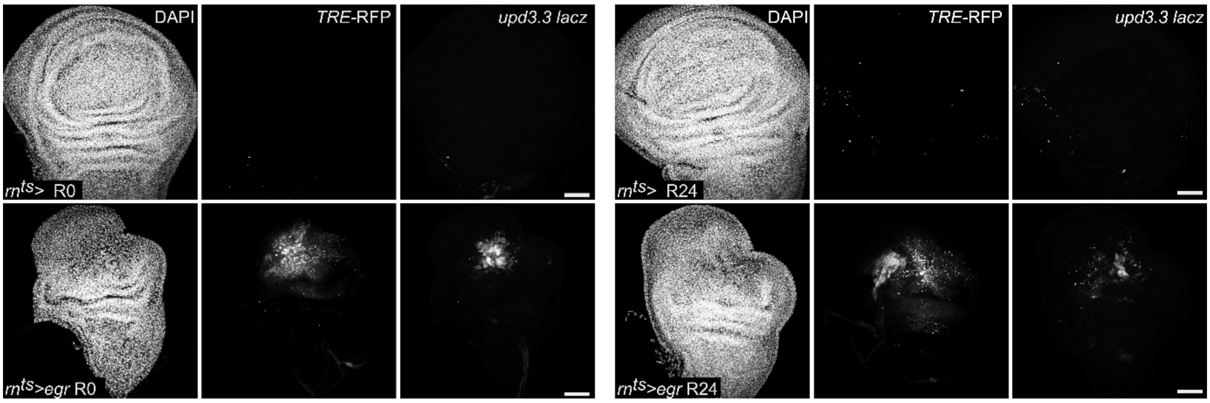

2. Related to this, in the current Eiger model, is there an 'end point' that can be analysed for a readout of successful regeneration? i.e. if cells in the JNK+ region do eventually die, can one look if surrounding STAT+ cells totally repopulate the area? Perhaps adult wings could be examined to reinforce the claims around the importance of these mutually exclusive domains? Later on, they could manipulate the JNK/JAK-STAT domains (e.g. using *Ptp61F* RNAi) and show images of the adult wings (the phosphatase RNAi should fail regeneration and adult wings should look abnormal)?

- Yes, there is an end point that can be analyzed for successful regeneration. We find that co-expression of *Ptp61F*-RNAi reduces the size of adult wings derived from formerly *egr*-expressing wing discs, suggesting that bistability is required for successful regeneration. We include this data now in the supplement to Fig. 7. Please note though, that the interpretation of the data is limited by (1) the long developmental time between imaginal disc damage and adult wing phenotype (several days), and (2) the adult wing phenotype could be disturbed by problems in epithelial sealing, regenerative growth, repatterning or differentiation. Thus, the exact link between an adult phenotype and regenerative processes is not always easy to discern.
- Importantly, not all JNK-positive cells eventually die. They can reenter the cell cycle when JNK/AP-1-signaling goes down. We can trace the cell population which is surviving reentry using *rn-GAL4>G-trace* for 24 h and 48 h after *egr*-expression ceases (R24 and R48). This experiment clearly shows that now also formerly high JNK-signaling cells contribute to repopulation of the central pouch domain. We included the data set now in Fig. 7.

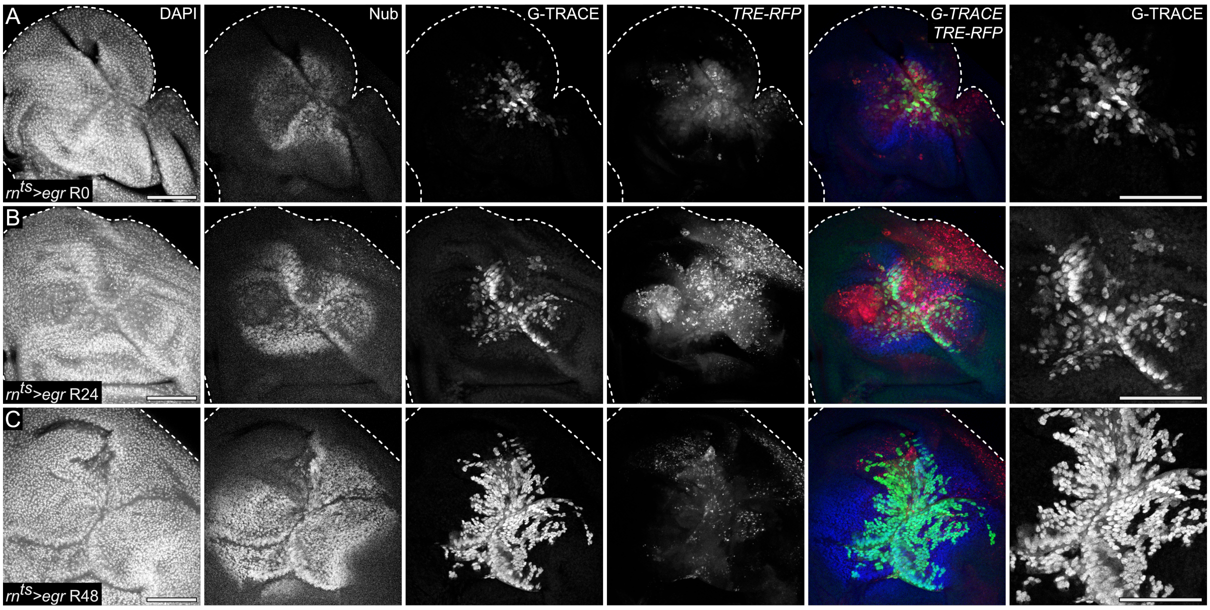

- We are not able to lineage-trace JAK/STAT activated cells directly. However, we can perform an analysis complimentary to tracing the JNK/AP-1 signaling cells by labeling the very peripheral hinge cells using GH146QF-QUAS tomato. The expressing cells locate primarily outside the JAK/STAT-activated population. 24 h into the recovery period after egr-expression (R24), some of these cells can be detected near the centre of the disc. However 24 h later (R48), most of these cells are again restricted to the very peripheral hinge. One could interpret this finding to mean that the JAK/STAT positive cells have repopulated the intervening domain. As this experiment does not represent a true lineage tracing experiment, we feel the interpretation is limited and only include these results in our response here.

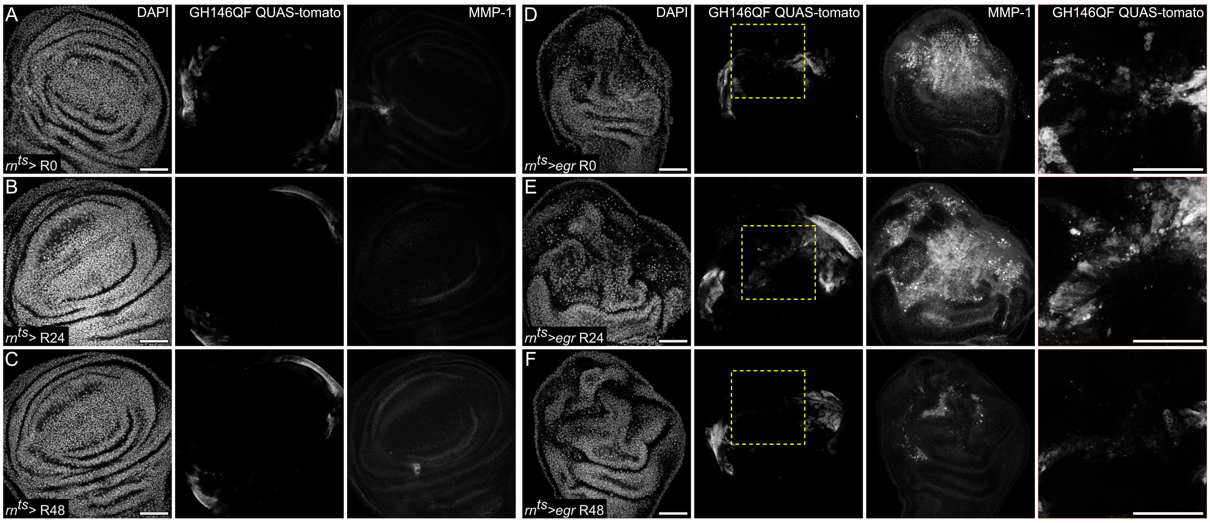

3. Related to point 1 above, is it known whether the mutual repression observed between JNK and JAK-STAT also occurs when the wing discs are damaged using an alternative method e.g. by forcing ectopic rpr expression (and does this also depend on expression of *Ptp61F*)?

- We find that ectopic expression of *rpr* kills the *rn-GAL4* expressing domain very, very quickly and more efficiently than *egr*-expression. This is likely due to the more direct activation of the caspase machinery by *rpr*, rather than via activation of pleiotropic JNK-signaling by *egr*. Almost no pouch cells can be detected after 24h of expression and consequently, the size of the JNK-activated death domain is small (see images below). While some of the preliminary data sets also suggest that bistability may exist, the optimization of this system to catch it at exactly the right moment of when *rpr*-expressing cells are still alive and activate JNK is difficult because the time between *rpr*-expression and death is so short.
- We thus feel that our focus on *egr*-expression and other JNK-activating genetic constructs, such as *hep*^{ACT} and *scrib-RNAi* are more useful to support our conclusion that JNK-signaling represses JAK/STAT.

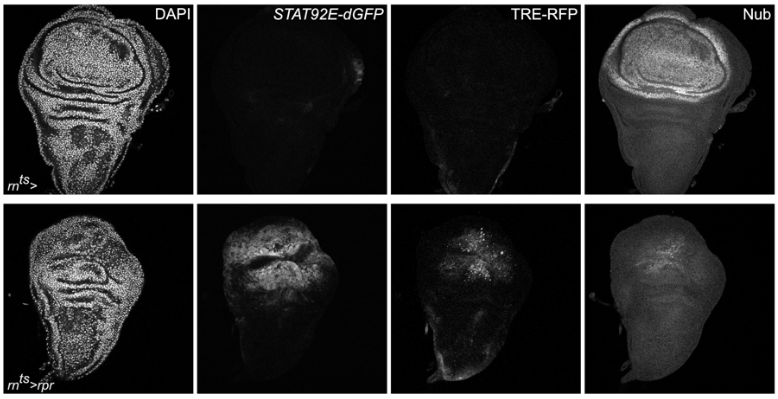

4. Have the authors tried making clones overexpressing Upd ligands to determine if this also induces similar JAK-STAT activity in surrounding cells? In other words, is secretion of Upds on their own sufficient to establish these mutually exclusive domains, or is JNK activation forcing JAK/STAT activation in neighbouring cells by other means?

- Some estimates of the different activities of the Upd ligands on surrounding cells can be derived from previous studies, for example [10-14]. We have not attempted to perform these experiments, as a genetic and fluorescence strategy for clone labeling, Upd1, 2 or 3 -labeling and pathway reporter activity was not systematically accessible. We feel that to really do this properly and test alternative non-autonomous activation mechanisms would be outside the focus and scope of this manuscript and revision. Furthermore, these results would not be central to our main novel conclusion that JNK/AP-1 represses JAK/STAT signaling cell-autonomously.

5. It would be interesting if the authors could comment on how far the Upd ligands are thought to travel from secreting cells in the JNK active domain to activate STAT. It appears that STAT is activated throughout most of the wing disc perhaps suggesting that Upds travel far? Or perhaps STAT cells participate in a positive feedback loop and make their own Upd which activates STAT in neighbouring cells? Positive feedback is included in the presented model, but it is unclear whether this is thought to be cell autonomous or non-cell autonomous amplification?

- Most of JAK/STAT activation occurs within a fairly restricted adjacent domain of hinge cells (please see Fig 3. I-K). This is consistent with the range of Upd1 'diffusion' from the source, as observed in egg chambers or other tissues (see [10-14]). Occasional activation in the notum may be due to low expression of *egr* in some *rn-GAL4* positive myoblasts.
- Positive feedback in JAK/STAT signaling cells is mainly mediated by upregulation of the receptor dome or the transcription factor STAT [11, 15-17], so it acts cell-autonomously (as included in the model). Upregulation of *upd* ligands in JAK/STAT signaling cells via a positive feedback loop has not been reported. We have also never observed evidence that JAK/STAT signaling cells upregulate *upd* ligands via a positive loop (see also Fig. 2D and Fig. 3A).

6. Figure 3: It would be interesting (although not essential for this revision) if the authors could use the mathematical model to further explore how the size of clone or the duration/intensity of JNK affects the signalling dynamics and mutual repression.

- We have performed a more extensive analysis of the model as suggested by reviewer 3. In this analysis we explore the relationship between egr, JNK activation and bistability. We find that there is no strict relationship between the size of the Eiger gradient and the intersection point of JAK/STAT and JNK (see Fig S5.1 I-K). However, the gradient width of JNK (Fig. S5-1), which

could serve as an indirect proxy for clonal size, and the activation rate of JNK (Fig. S5-2) are central parameters that determine bistable patterns.

7. For the *Ptp61F* knockdown experiments in Figure 4, it is important that the authors can demonstrate that the same phenotype can be achieved with multiple independent RNAi lines to preclude off target effects. Ideally the authors would also show (although it is not essential) the effectiveness of the RNAi knockdown (e.g. using RT-qPCR).

- In our hands, only one of the reported RNAi constructs is passing our control assays (upregulation of developmental JAK/STAT signaling in the hinge, see Fig S6.3 G-I) AND induces JAK/STAT derepression in *egr*-expressing discs. This line has been previously shown to be very effective at knocking down Ptp61F even at the protein level, if expressed by itself [18].
- We tested other RNAi lines provided by VDRC or BDSC but they do not visibly affect *egr*-driven JAK/STAT repression. For some of these lines a partial knockdown of transcripts had been previously confirmed after expression of these constructs for 2 days or longer. Some of these lines have been previously used in the context of UAS-dicer, which likely increases effectiveness [19][20]. We considered to include UAS-Dicer in our genotypes but because of all the required transgenes are located on chromosome 2 or 3 (*rn*>*egr*, *TRE-RFP*, *stat-GFP*, UAS-RNAi, UAS dicer and so on) we would have to devise recombination strategies.
- Since we use a line that has been confirmed to effectively reduce Ptp61F protein levels, and it derepresses developmental JAK/STAT activity patterns, we feel confident that this line works. Since our experiments are limited by a relatively short expression for just 24 h, we interpret the lack of JAK/STAT derepression by other constructs to be due to partial or ineffective reduction in Ptp61F levels within the short expression window of just 24 h. Since we cannot change these conditions and given the limitations on genetic Dicer strategies, we kindly ask you to reconsider your request.

8. To fully describe the system, it is important to analyse the expression of *Ptp61F*. For example, can in situ hybridisation be used to show i) that *Ptp61F* is induced only in the JNK compartment and ii) explore the timeline of *Ptp61F* induction (in control vs *egr* expressing discs). Alternatively, two EGFP-FlAsH-StrepII-TEV-3xFlag tagged *Ptp61F* lines are available from Bloomington which could reveal Otp61F levels.

- We tested the two EGFP-FlAsH-StrepII-TEV-3xFlag tagged *Ptp61F* lines. However, a close inspection of the sequencing data provided by Flybase actually reveals that these constructs are inserted in the wrong direction. These lines do not show any significant GFP expression or GFP-regulation (in our hands). We thus think that they are not true GFP-tagged *Ptp61F* lines.
- We also obtained multiple (older) anti-*Ptp61F* antisera from Carolyn Worby. However, we could never conclusively prove that it detected overexpression or knock down of *Ptp61F*, so we are not confident that the sera still work.
- We now present data from a single cell RNA-seq study on *egr*-expressing discs [21] showing that *Ptp61F* is mildly elevated in surviving, *egr*-expressing cells. Importantly, we now also provide evidence that also *Socs36E* is repressing JAK/STAT in JNK-signaling cells (new Fig. 6 and S6-3) and that *Socs36E* is induced in *egr*-expressing disc (Fig. S6-3).

9. The data presented demonstrates that loss of *Ptp61F* can elevate STAT activity, but is over-expression *Ptp61F* also sufficient to stop activation of JAK STAT? A UAS-*Ptp61F* transgenic line is available from Bloomington so the authors should be able to perform this experiment .

- We tested a UAS-line generated by Martin Zeidler, which expresses the predicted nuclear isoform of *Ptp61F* (w; p[w+, UAS-*Ptp61F*c]5a.3/TM3) that we would hypothesize to act on *Stat92E*. We find that overexpression of this isoform using *en-GAL4* in preliminary experiments appears to be lethal as we cannot recover any larvae. Because of this lethality, we did not pursue this line of investigation. In addition, the next logical experiments with these constructs would have taken us back into the *egr,Stat92-HA*-system. Given the risk that the observed lethality imposes on the interpretation of *egr,Stat92-HA*-rescue experiment, we did not develop a recombination genotype for all our constructs on the 3rd chromosome (the *egr*-expression system, the UAS-*Ptp61F* and UAS- *egr,Stat92-HA*-are all on chromosome 3). The time frame for generating and testing such a recombinant chromosome, and performing a well-controlled, quantified experiment was outside the possibilities of this revision.

10. In the experiments to demonstrate that *Ptp61F* inhibits JAK-STAT activity via dephosphorylation of STAT in which STAT92 is over-expressed to saturate the *Ptp61F* dephosphorylase activity, is it possible that STAT92 over-expression would increase JAK-STAT activity (in the presence of egr) even if unrelated to *Ptp61F*? Indeed it appears that as rn>*Stat92E* alone causes a modest increase of JAK/STAT signalling. To confirm the role of *Ptp61F*, can transgenic modified STAT constructs be used such as one in which STAT cannot be dephosphorylated? Alternatively, if this experiment is genetically too difficult to perform, perhaps the authors could expose the discs to Tyrosine phosphatase inhibitors?

- As we removed the relevant conclusions (Ptp61F directly acting on *Stat92E*) from the manuscript, we hope that these revisions are not central to the manuscripts anymore.
- However, we still looked into these experiments: The relevant construct would most likely have to be a STAT Y704 phosphomimic. Such a construct does not exist (only a phospho-ablative construct for this residue).
- Chemical PTP1B inhibitors exist. If they work on *Drosophila Ptp61F* is unknown. In addition, we find that disc viability, proliferation and signaling is strongly impaired in published protocols of wing imaginal disc culture (see for example [22]), which we would have to use to perform this experiment. In addition, the inhibitor could not be spatially targeted to JNK-signaling cells but would act on the entire tissue. Because of these significant limitations and extensive controls/optimization required to draw reliable conclusions, we feel that such an approach is outside the time frame of this revision.

11. Figure 6: Does *Ptp61F* expression downstream of JNK signalling also repress STAT activity in the tumour model?

- We performed a single preliminary experiment and could not observe a visible growth phenotype in the Ptp61F RNAi co-expressing tumor discs under standard experimental conditions (no other markers examined). Due to the time required in properly assessing any changes and optimizing the experimental conditions accordingly, and due to genetic incompatibility of all 7 required constructs (ptp61F RNAi; RasV12; scribRNAi; rn>; tub-GAL80ts; stat-GFP;TRE-RFP) making complex recombination strategies necessary to properly assess the signaling state of disc as well, we stopped this line of investigation.

12. The model presented in Figure 7 is interesting but would benefit from a more thorough description of when 'transient or low level' JNK levels might occur. Given that in the eiger overexpression wing disc model it takes 24h for full mutual suppression between JNK and STAT, it will be interesting to know how a more transient burst of JNK activity affects STAT?

- We restructured the model and toned-down our story line on transient vs chronic activation. Given the comments by all reviewers, we felt that this point got a little lost and we thought it would be better to really focus the manuscript on the essential messages.
- However, the initial 'transient or low level' quote referred to the 7h and 14h expression 'bursts' of *egr* (now Fig. 3) or expression of *scrib-RNAi* (now Fig. S4.1 G).

Minor comments

1. In Figure 2A-B, the authors use expression of p35 to prevent cell death in the 'hep-active' discs. It is a little difficult to observe the details of the clones (and surrounding STAT activity) - the Figure would be improved by inclusion of higher magnification insets. It could also be beneficial to include an additional control here - staining in wild type wing discs where there is no ectopic p35 expression.

- We have worked a lot with p35 and have not observed phenotypes after 24 h of expression in clones. We thus feel that these clones are the more relevant controls to really demonstrate that p35 alone does not have an effect. We also do not want to further expand the already extensive supplemental data set. So, we kindly ask you to reconsider the idea to also include the additional control of just wild type clones.
- We rearranged the figure and hope that the little bit larger images help to improve visibility of the data.

2. Figure 2K - is this STAT activity not DAPI? And if not, please show low mag images of STAT throughout wing disc over time.

- Yes indeed. It is now corrected. We also rearranged the figure and hope that the images are now better suited to assess the data.

3. Please include more detail on previous work with Zfh2 - for example, what are the consequences of RNAi Zfh2 in JAKSTAT domain and elevation of JNK?

- Based on comments by another reviewer we removed this data from the manuscript to more strongly focused on repression of JAK/STAT by JNK. Further refining the datatsets for Zfh2 was not central to our main message.
- However, our previous data suggests that overexpressing Zfh2 in JNK-signaling cells increases the size of the surviving *rn-GAL4-egr* population [9], likely via downregulation of JNK (first manuscript draft, Fig. S3A-C).

4. Figure S2K - please explain in more detail how the region of interest was quantified?

- We replaced this figure with more intuitive quantifications of JAK/STAT reporter activity (Fig 3M and Fig. S3.2 F).

5. Please show evidence MMP1 upregulated by JNK.

- It is well-established that JNK/AP-1 signaling targets MMP-1, (for example AP-1 motifs can be detected in the 5'UTR of MMP-1 gene, see [23]). MMP-1, in our hands, is a robust marker of TRE-RFP activation domains (please see our previous work in [2]: https://iiif.elifesciences.org/lax/41036%2Felife-41036-resp-fig4-v2.tif/full/full/0/default.jpg).

6. In Figure 4 F-J, it is unclear why the discs were examined at 12h post induction when all other experiments use 7h, 14 and 24h.

- This image is just a control to demonstrate that UAS-*Stat92E* works. However, at 12 h expression the disc is at a younger developmental stage and developmentally regulated expression of Upd is higher. Reporter activation is thus easier to see. We now explain this in the figure legend.

7. In Figure 5, there appears to be considerable apoptosis (via Dcp1 staining) in *egr* expressing discs and these spread across most of the disc when STAT92 is also expressed. Could the authors suggest how these cells (originating from the JNK+ domain) might spread so far across disc?

- We suggest that this is just a consequence of space limitations. If apoptotic cells are extruded basally, their debris is ‘squeezed’ in between the basal surface and the basement membrane and may thus distribute to more distant sites if many cells die at once. If apoptotic cells are extruded apically (which can happen in *egr*-expressing discs) they are extruded into the disc lumen and their remains can then be ‘moved’ around within the lumen to distant sites.

References

1. Crucianelli C, Jaiswal J, Vijayakumar Maya A, Nogay L, Cosolo A, Grass I, et al. Distinct signaling signatures drive compensatory proliferation via S-phase acceleration. PLoS Genet. 2022;18(12):e1010516. Epub 20221215. doi: 10.1371/journal.pgen.1010516. PubMed PMID: 36520882.

2. Cosolo A, Jaiswal J, Csordas G, Grass I, Uhlirova M, Classen AK. JNK-dependent cell cycle stalling in G2 promotes survival and senescence-like phenotypes in tissue stress. Elife. 2019;8. Epub 20190208. doi: 10.7554/eLife.41036. PubMed PMID: 30735120; PubMed Central PMCID: PMCPMC6389326.

3. Cohen E, Allen SR, Sawyer JK, Fox DT. Fizzy-Related dictates A cell cycle switch during organ repair and tissue growth responses in the Drosophila hindgut. Elife. 2018;7. Epub 20180817. doi: 10.7554/eLife.38327. PubMed PMID: 30117808; PubMed Central PMCID: PMCPMC6130973.

4. Tamori Y, Deng WM. Tissue repair through cell competition and compensatory cellular hypertrophy in postmitotic epithelia. Dev Cell. 2013;25(4):350-63. doi: 10.1016/j.devcel.2013.04.013. PubMed PMID: 23685249; PubMed Central PMCID: PMC3891806.

5. Losick VP, Fox DT, Spradling AC. Polyploidization and cell fusion contribute to wound healing in the adult Drosophila epithelium. Curr Biol. 2013;23(22):2224-32. Epub 20131031. doi: 10.1016/j.cub.2013.09.029. PubMed PMID: 24184101; PubMed Central PMCID: PMCPMC3898104.

6. Xiang J, Bandura J, Zhang P, Jin Y, Reuter H, Edgar BA. EGFR-dependent TOR-independent endocycles support Drosophila gut epithelial regeneration. Nat Commun. 2017;8:15125. Epub 20170509. doi: 10.1038/ncomms15125. PubMed PMID: 28485389; PubMed Central PMCID: PMCPMC5436070.

7. Jiang H, Patel PH, Kohlmaier A, Grenley MO, McEwen DG, Edgar BA. Cytokine/Jak/Stat signaling mediates regeneration and homeostasis in the Drosophila midgut. Cell. 2009;137(7):1343-55. Epub 2009/07/01. doi: 10.1016/j.cell.2009.05.014. PubMed PMID: 19563763; PubMed Central PMCID: PMCPMC2753793.

8. Ruiz-Losada M, Gonzalez R, Peropadre A, Gil-Galvez A, Tena JJ, Baonza A, et al. Coordination between cell proliferation and apoptosis after DNA damage in Drosophila. Cell Death Differ. 2021. Epub 20211125. doi: 10.1038/s41418-021-00898-6. PubMed PMID: 34824391.

9. La Fortezza M, Schenk M, Cosolo A, Kolybaba A, Grass I, Classen AK. JAK/STAT signalling mediates cell survival in response to tissue stress. Development. 2016;143(16):2907-19. Epub 20160706. doi: 10.1242/dev.132340. PubMed PMID: 27385008.

10. Borensztejn A, Boissoneau E, Fernandez G, Agnes F, Pret AM. JAK/STAT autocontrol of ligand-producing cell number through apoptosis. Development. 2013;140(1):195-204. doi: 10.1242/dev.079046. PubMed PMID: 23222440.

11. Hombria JC, Brown S, Hader S, Zeidler MP. Characterisation of Upd2, a Drosophila JAK/STAT pathway ligand. Dev Biol. 2005;288(2):420-33. Epub 20051107. doi: 10.1016/j.ydbio.2005.09.040. PubMed PMID: 16277982.

12. Vollmer J, Fried P, Aguilar-Hidalgo D, Sanchez-Aragon M, Iannini A, Casares F, et al. Growth control in the Drosophila eye disc by the cytokine Unpaired. Development. 2017;144(5):837-43. doi: 10.1242/dev.141309. PubMed PMID: 28246213.

13. Osman D, Buchon N, Chakrabarti S, Huang YT, Su WC, Poidevin M, et al. Autocrine and paracrine unpaired signaling regulate intestinal stem cell maintenance and division. J Cell Sci. 2012;125(Pt 24):5944-9. Epub 20121004. doi: 10.1242/jcs.113100. PubMed PMID: 23038775.

14. Wright VM, Vogt KL, Smythe E, Zeidler MP. Differential activities of the Drosophila JAK/STAT pathway ligands Upd, Upd2 and Upd3. Cell Signal. 2011;23(5):920-7. Epub 20110122. doi: 10.1016/j.cellsig.2011.01.020. PubMed PMID: 21262354.

15. Shlevkov E, Morata G. A dp53/JNK-dependant feedback amplification loop is essential for the apoptotic response to stress in Drosophila. Cell death and differentiation. 2012;19(3):451-60. doi: 10.1038/cdd.2011.113. PubMed PMID: 21886179; PubMed Central PMCID: PMC3278728.

16. Khan SJ, Abidi SNF, Skinner A, Tian Y, Smith-Bolton RK. The Drosophila Duox maturation factor is a key component of a positive feedback loop that sustains regeneration signaling. PLoS Genet. 2017;13(7):e1006937. Epub 2017/07/29. doi: 10.1371/journal.pgen.1006937. PubMed PMID: 28753614; PubMed Central PMCID: PMCPMC5550008.

17. Pinal N, Martin M, Medina I, Morata G. Short-term activation of the Jun N-terminal kinase pathway in apoptosis-deficient cells of Drosophila induces tumorigenesis. Nat Commun. 2018;9(1):1541. Epub 2018/04/20. doi: 10.1038/s41467-018-04000-6. PubMed PMID: 29670104; PubMed Central PMCID: PMCPMC5906466.

18. Ku HY, Wu CL, Rabinow L, Chen GC, Meng TC. Organization of F-actin via concerted regulation of Kette by PTP61F and dAbl. Mol Cell Biol. 2009;29(13):3623-32. Epub 20090427. doi: 10.1128/MCB.00229-09. PubMed PMID: 19398577; PubMed Central PMCID: PMCPMC2698756.

19. Saadin A, Starz-Gaiano M. Identification of Novel Regulators of the JAK/STAT Signaling Pathway that Control Border Cell Migration in the Drosophila Ovary. G3 (Bethesda). 2016;6(7):1991-2002. Epub 20160707. doi: 10.1534/g3.116.028100. PubMed PMID: 27175018; PubMed Central PMCID: PMCPMC4938652.

20. La Marca JE, Willoughby LF, Allan K, Portela M, Goh PK, Tiganis T, et al. PTP61F Mediates Cell Competition and Mitigates Tumorigenesis. Int J Mol Sci. 2021;22(23). Epub 20211125. doi: 10.3390/ijms222312732. PubMed PMID: 34884538; PubMed Central PMCID: PMCPMC8657627.

21. Floc'hlay S, Balaji R, Christiaens V, González-Blas CB, De Winter S, Hulselmans G, et al. Shared enhancer gene regulatory networks between wound and oncogenic programs. Biorxiv. 2022. doi: 10.1101/2022.06.17.496596.

22. Balaji R, Bielmeier C, Harz H, Bates J, Stadler C, Hildebrand A, et al. Calcium spikes, waves and oscillations in a large, patterned epithelial tissue. Sci Rep. 2017;7:42786. Epub 20170220. doi: 10.1038/srep42786. PubMed PMID: 28218282; PubMed Central PMCID: PMCPMC5317010.

23. Uhlirova M, Bohmann D. JNK- and Fos-regulated Mmp1 expression cooperates with Ras to induce invasive tumors in Drosophila. Embo J. 2006;25(22):5294-304. PubMed PMID: 17082773.
