# Supplementary material for: Mutual repression between JNK/AP-1 and JAK/STAT stratifies senescent and proliferative cell behaviors during tissue regeneration
Source: PLoS Biol. 2023 May 30;21(5):e3001665. doi: 10.1371/journal.pbio.3001665 (PMC10228795; doi:10.1371/journal.pbio.3001665)
Supplement: Attachment — Submitted filename: 001 Responses to Reviewers.pdf [file pbio.3001665.s017.pdf]

Responses to the reviewers

Rev. 1: Andrea Page-McCaw – note that this reviewer has signed her review
The authors have fully addressed my comments and concerns. The bistable self-organizing patterning of JNK and JAK/STAT is an interesting and important finding, and this paper is suitable for publication.

Rev. 2:
Jaiswal and colleagues report mutually exclusive activities of JNK and JAK/STAT during regeneration and tumor growth in Drosophila wing discs. The revision addresses my concerns from the first round of review except for the last comment (specific concern #8) that the authors asked clarification for. I will try to provide that here.

In figure 3 of the original manuscript and figure 5 of the revised version, the authors show two different models. In the first model, JNK activates JAK/STAT via Upds but then JAK/STAT represses JNK (unidirectional repression). In the second, initial activation of JAK/STAT is followed by later repression of JAK/STAT by JNK (bidirectional repression). My concern is that even if unidirectional model is the correct one, as JNK-active cells die, they cannot produce Upds to activate JAK/STAT, leading to loss of JAK/STAT activation. This would then look as if initial activation of JAK/STAT is followed by repression of JAK/STAT (mutual repression model) when what is happening is not repression but loss of activation. The modeling does not include complications due to the death of JNK-active, Upd-producing cells over time. Can this be ruled out? I suspect that response to my General concern 2 (the role of cell death in JAK/STAT activation) may already have addressed this specific
concern.

- In the periphery, JAK/STAT is normally activated by the non-autonomous action of secreted cytokines from JNK-signaling cells in the pouch. If the unidirectional model was true and the JNK-signaling cell died and thus did not produce cytokines anymore, then JAK/STAT activation <u>outside</u> the JNK signaling domain in the periphery should also be downregulated. As this is clearly not the case and JAK/STAT signaling outside the JNK domain stays high in all conditions tested (see Fig 3 and Fig 4), we think that we have strong evidence that cytokines continue to be secreted from the JNK-signaling cells in the center, which maintain JAK/STAT signaling high in the periphery. This means that there is no loss of activation JAK/STAT activation due to death of JNK-signaling cells. Rather, the surviving population continues to produce upd cytokines.

Rev. 3:
The authors have significantly revised and improved their manuscript. They provide a more detailed description of the experimental system, of the model and of their strategy of exploration of the parameter space. They also provide representative plots of the temporal dynamics of their model and additional analysis.

They have decided to not compare the time-evolution of their model with the experimental results, which I think is a missed opportunity. They refer to the fact that "while we have a fairly good understanding of the behavior of our reporters, we did not vigorously determine their (linear) activity ranges, so it would be naive to correlate fluorescence levels with precise mathematical activities (at this point in time)." On the other hand, they do use those fluorescence levels to analyze spatial patterns, so they are quite confident on those reporters. Nevertheless, the scope of the manuscript is

authors' choice. I still request the authors to quantify the pattern of JNK and JAK/STAT activation as a function of distance from the center at different time-points, using the data reported in Fig.1 and Fig. 3, unless they have already done it and I missed it.

I do not think that they have addressed my last question on the effects of activation of JAK/STAT at the center of the disc and JNK/AP-1 at its periphery on apoptosis. They have commented on the reciprocal effects of JNK and JAK/STAT, but not on its effect on apoptosis. I request the authors to comment on the effects of this perturbation.

I think that, once implemented these minor revisions, the manuscript will be acceptable for publication.

- We truly did not mean to be dismissive of the idea to quantify reporter activities *per se*. We meant to say that we understand the reporters well enough to be able to say that they are 'not', 'mildly' or 'strongly' upregulated (or downregulated). However, we do not currently understand what a 20% or 80% increase in fluorescence means within respect to real biological signaling intensity, and much less so in the context of a modelling approach with multiple differential equations and many unknown variables (see Fig S5.2). We therefore think that the dynamics of the reporters and many other signaling components would have to be quantified to interpret quantitative changes and their exact functional effect on signaling dynamics.

- However, we have quantified the imaging data in Figure 3 to illustrate both temporal dynamics and spatial distribution of JNK/AP-1 and JAK/STAT signaling. We now provide these data sets in new Figure 3L-O and new Supp Figure S3.2. The results are completely consistent with our previous conclusions.

- We think that excess apoptosis observed by co-activation of JAK/STAT and JNK/AP-1 is a cell-autonomous response. We do not think that excess apoptosis is due to a more global effect from perturbed pattern. In Fig S7 D,E we actually show that all apoptosis arises from cells with co-activation of JAK/STAT and JNK/AP-1 and not from another cell population that may be affected by the changes in spatial pattern. Moreover, when we change the pattern of JNK-activation, we only observed apoptosis in domains targeted by the GAL4 driver which activates JNK (for example conditions in Fig 3H or S3.2 A,B). We did not observe apoptosis outside the JNK-activated domain, which may have arisen from non-autonomous effects of an altered spatial patterned of JNK and JAK/STAT activation.

Rev. 4:
We thank the authors for the additional experiments/quantification provided in this revised version of the manuscript. Whilst the authors have provided additional data to address some of our original comments and explained why some of the additional experiments we had requested are not feasible, we still have the following concerns that we feel should be addressed (and should be relatively straight forward to address) before final publication:

1. Regarding levels of Ptp61F:
We requested more information on the expression of Ptp61F, including the timing of expression and whether expression is restricted to the egr-expressing (JNK positive) domain. The authors refer to scRNA-seq data to help address this question (supplemental figure S6.3). However in these scRNAseq graphs, it appears as if egr expression actually induces Ptp61F (and the new additional

candidate Socs36E) throughout the disc, rather than just in the rn-expressing domain. Some additional quantification using this scRNAseq data would be important to confirm how the expression of these key factors change following egr induction (within and outside the rn positive domain). These new scRNAseq data also suggest that Ptp61F and Socs36E induction might restrain JAKSTAT signalling throughout the disc, rather than in just the JNK positive domain? A comment on this would be useful.

- *Ptp61F and socs36E* are significantly induced in the JNK-signaling cells identified in the scRNA-Seq study (see ‚beta' cluster signatures below which is already published in Supp Table 3 in (Floc'hlay, Balaji et al. 2022) and cited in our manuscript). We state this fact now more directly in the manuscript.

	Log2FC	Adjusted_pval	mean_expr	perc_expr	contrast
**Socs36E**	2.7336655	3.3268196403999e-20	1.9643514156341553	0.9875	wound_beta_versus_control_pouch
**Ptp61F**	1.6718576	0.00013578650471289568	0.9843866229057312	0.55	wound_beta_versus_control_pouch

- We use *rn* as a marker for the visualization of cells in Fig S6.3, which are targeted by our *rn-GAL4>egr* system to induce JNK-signaling. Upregulation of both *ptp61F and socs36E* transcripts in cells outside the *rn*-positive population is absolutely possible. We have repeatedly described JNK-activation outside the *rn-GAL4, egr*-expressing cell population (for example (Cosolo, Jaiswal et al. 2019)). So, yes, *ptp61F* and *socs36E* may be induced more widely in *egr*-expressing discs. Importantly, we show that JNK-signaling cells, the focus of our story, functionally depend on these two components to suppress JAK/STAT signaling. We did not investigate if *ptp61F* and *socs36E* have functions in other cell populations, which is certainly possible but beyond the scope of this paper.

2. Are the JNK positive cells quiescent or senescent:
The authors state that JNK positive cells undergo senescence. However, they now also provide additional data to show that these JNK cells actually re-enter the cell cycle and re-populate a significant portion of the disc after JNK activity subsides (G-trace experiments). This raises the question whether these cells are senescent or whether they are rather becoming temporarily quiescent?

- While JNK-signaling cells do not become terminally senescent, these cells display all cellular markers (this manuscript) and transcriptional programs (Floc'hlay, Balaji et al. 2022) described for terminal senescence in mammalian systems (Hernandez-Segura, Nehme et al. 2018, Lopes-Paciencia, Saint-Germain et al. 2019). We thus decided to call these JNK-signaling cells senescent, consistent with the recent literature on transient senescent phenomena in development and wound healing (Calcinotto, Kohli et al. 2019). Moreover, other peripheral tissues such as the notum or the antennal disc are also reducing their proliferation upon pouch damage, yet these peripheral cells do not display any of the same senescent markers as JNK-signaling cells (unpublished). We apply the label 'quiescent' to these peripheral cells, as they also become less metabolically active (due to reduced insulin-secretion from IPS cells). To really semantically distinguish and discuss these two differently arrested populations, and to highlight the significance of a transient senescent programs, we chose to apply this terminology.

3. Image quantification:
As pointed out by another Reviewer there were some concerns with quantification in the original manuscript. Related to point (1) above, given the paper's core concept surrounds the establishment of 2 separate signalling domains, there is a lack of quantification of key proteins (including levels of upd,

ptp16F and socs36E) in these 2 separate domains (within the same egr expressing discs). The majority of quantification shows that levels of upd, ptp61f etc change over time following egr induction, but this is compared with control discs. What is missing is the quantification of the spatial distribution of these proteins in the 2 domains following egr induction.

Moreover in Figure 3M: it is unclear where the measurements of Jak/STAT92 activation have been taken. In the main text, the authors refer to 2 subdomains within the disc (pouch and hinge) but it is unclear whether the quantifications of JAK/STAT intensity reported in the graph come from the pouch, the hinge or both.As over the course of 24h the levels changes in the two domains, maybe it would be better to show the two quantifications separately rather than showing a cumulative values?

- We have now quantified the imaging data in Figure 3 to illustrate both temporal dynamics and spatial distribution of JNK/AP-1 and JAK/STAT signaling in the pouch and periphery (hinge). We now provide these data sets in new Figure 3L-O and new Supp Figure S3.2. We furthermore adapted the schemes, figure legends and methods to be more precise about where the data points were measured. The obtained results are completely consistent with our previous conclusions.

- Spatial distribution for many other pathway components was unchanged (see Fig S6.1 and S6.2). As these data sets yielded negative results in our hands, we did not quantify this unchanged distribution of pathway components. To provide some measure of reproducibility, we had already noted the number of discs examined for each condition in the figure legends.

- We cannot quantify Socs36E and Ptp61F at the protein level, due to lack of tools. However, we had already provided some measure of spatial description via scRNA-seq (see discussion to point 1).

References

Calcinotto, A., J. Kohli, E. Zagato, L. Pellegrini, M. Demaria and A. Alimonti (2019). "Cellular Senescence: Aging, Cancer, and Injury." Physiol Rev **99**(2): 1047-1078.

Cosolo, A., J. Jaiswal, G. Csordas, I. Grass, M. Uhlirova and A. K. Classen (2019). "JNK-dependent cell cycle stalling in G2 promotes survival and senescence-like phenotypes in tissue stress." Elife **8**.

Floc'hlay, S., R. Balaji, V. Christiaens, C. B. González-Blas, S. De Winter, G. Hulselmans, M. De Waegeneer, X. Quan, D. Koldere, M. Atkins, G. Halder, A.-K. Classen and S. Aerts (2022). "Shared enhancer gene regulatory networks between wound and oncogenic programs." Biorxiv.

Hernandez-Segura, A., J. Nehme and M. Demaria (2018). "Hallmarks of Cellular Senescence." Trends Cell Biol **28**(6): 436-453.

Lopes-Paciencia, S., E. Saint-Germain, M. C. Rowell, A. F. Ruiz, P. Kalegari and G. Ferbeyre (2019). "The senescence-associated secretory phenotype and its regulation." Cytokine **117**: 15-22.
