# Supplementary material for: Mutual repression between JNK/AP-1 and JAK/STAT stratifies senescent and proliferative cell behaviors during tissue regeneration
Source: PLoS Biol. 2023 May 30;21(5):e3001665. doi: 10.1371/journal.pbio.3001665 (PMC10228795; doi:10.1371/journal.pbio.3001665)
Supplement: Attachment — Submitted filename: 004 Response.docx [file pbio.3001665.s018.docx]

**Requests**

DATA POLICY:

Many thanks for submitting the data underlying the graphs shown in the figures. I have checked them all and I am missing the data for the following figures:

Fig. 3C, F, G; Fig. 5B-D, B’-D’, B’’-D’’; Fig. S3.1E-G, K-M; Fig. S5.1A-G, G’, G’’, H-K and Fig. S5.2

In addition, please ensure that figure legends in your manuscript include information on WHERE THE UNDERLYING DATA can be found, and ensure your supplemental data file/s has a legend.

Please ensure that your Data Statement in the submission system accurately describes where your data can be found.

-------------------------------------------------------------------------
BLOT AND GEL REPORTING REQUIREMENTS:

We require the original, uncropped and minimally adjusted images supporting all blot and gel results reported in an article's figures or Supporting Information files. We will require these files before a manuscript can be accepted so please prepare and upload them now. Please carefully read our guidelines for how to prepare and upload this data: <https://journals.plos.org/plosbiology/s/figures#loc-blot-and-gel-reporting-requirements>

**Response**

We have assembled the requested source data in the following files:

**File S1 Source data for all experimental work**

Source data for all quantifications derived from experimental work presented in this study (Fig 1-8).

**File S2 Source data for mathematical modeling Fig 5**

Source data for graphs derived from mathematical modeling presented in Fig 5.

**File S3 Source data for mathematical modeling Fig S5.1**

Source data for graphs derived from mathematical modeling presented in Fig S5.1.

**File S4 Source data for mathematical modeling Fig S5.2**

Source data for graphs derived from mathematical modeling presented in Fig S5.2.

**File S5 Source data for Western blots in Fig S6.1**

Original Western blot images for data shown in Fig S6.1 D.

We reference these files in the relevant figure legends.

We are not sure how helpful the source data in **Files S2, S3, and especially File S4** truly are. They contain the raw data points from mathematical modeling for all 2D plots (time/space) or (conc./counts) and thus contain an extensive amount of information. We await your advice on whether to include them (or not).
